# The effects of HIV self-testing on the uptake of HIV testing, linkage to antiretroviral treatment and social harms among adults in Africa: A systematic review and meta-analysis

**DOI:** 10.1371/journal.pone.0245498

**Published:** 2021-01-27

**Authors:** Bernard Njau, Damian J. Damian, Leila Abdullahi, Andrew Boulle, Catherine Mathews

**Affiliations:** 1 School of Public Health and Family Medicine, University of Cape Town, Cape Town, South Africa; 2 Save the Children International SCI, Somalia/Somaliland Country Office, Nairobi, Kenya; 3 Health Systems Research Unit, South African Medical Research Council, Cape Town, South Africa; Makerere University School of Public Health, UGANDA

## Abstract

**Introduction:**

HIV infection is still a global public health problem. More than 75% of HIV-infected people are in Africa, and up to 54% are unaware of their HIV status, limiting access to antiretroviral treatment.

**Context and purpose of the study:**

This review aimed to determine whether HIV self-testing (HIVST) increases the uptake of testing, the yield of new HIV-positive diagnoses, and the linkage to antiretroviral treatment, and social harms among adults in Africa.

**Methods:**

PubMed, The Cochrane Central Register of Controlled Trials (CENTRAL), Pan African Clinical Trials Registry, The Cochrane Database of Systematic Reviews (CDSR), Databases of Abstracts of Reviews of Effectiveness (DARE), Social Sciences Citation Index, Web of Science and African Index Medicus databases were searched from 1998 to 2019 (updated in December 2019). Eligible trials employed randomized controlled trials (RCTs), before/after studies, and interrupted time series design comparing HIVST to standard HIV testing services or comparing different approaches to HIVST among adults living in Africa were systematically sought.

**Results:**

After searching 2,617 citations eleven trials were identified including 59,119 participants from four (4) African countries. Meta-analysis of seven trials showed a significant increase in the uptake of HIVST compared to standard HIV testing services: Both fixed-effects (Rate Ratio (RR) = 2.64, 95% CI: 2.51 to 2.79), and random-effects (RR) = 3.10, 95% CI: 1.80 to 5.37, and a significant increase in the uptake of couples’ HIVST (RR = 2.50, 95% CI: 2.29 to 2.73 in fixed-effects models; and RR = 2.64, 95% CI: 2.01 to 3.49 in random-effects model). A decrease in linkage to care and ART was observed in HIVST compared to standard HIV testing services (RR = 0.88, 95% CI: 0.88 to 0.95 in fixed-effects models; and RR = 0.78, 95% CI: 0. 56 to 1.08 in random-effects models). Six RCTs measured social harms, with a total of ten reported cases related to HIVST. One RCT comparing two approaches to HIVST showed that offering home-based HIVST with optional home-initiation of antiretroviral treatment increased the reporting of a positive HIV test result (RR: 1.86; 95% CI: 1.16 to 2.98), and linkage to antiretroviral treatment (RR: 2.94; 95% CI: 2.10 to 4.12), compared with facility-based linkage to antiretroviral treatment.

**Conclusions:**

HIVST has the potential to increase the uptake of HIV testing compared to standard HIV testing services. Offering HIVST with optional home initiation of HIV care compared to HIVST with facility-based HIV care increases HIV positivity and linkage to antiretroviral treatment. Reported incidences of intimate partner violence related to HIVST were rare. Future research should focus on the potential of HIVST to reach first-time testers, the effect of using different approaches to HIVST, and strategies for linkage to HIV services.

**Systematic review registration:**

This systematic review was prospectively registered on the Prospero International Prospective Register of Systematic Review (CRD42015023935).

## Introduction

Globally, an estimated 35.0 million people are living with HIV, with more than 2.1 million [1.9 million–2.4 million] new HIV infections and 1.5 million deaths in 2013 [1]. Africa remains the most affected region, contributing more than two-thirds of the global burden of HIV [2]. The UNAIDS/WHO has set a “95-95-95” global target to be reached by 2030. The global target stipulates that 95% of adults will know their HIV status, 95% of HIV positive will receive sustained antiretroviral treatment (ART), and 95% of those who are on ART will achieve viral load suppression by 2030 [3].

In Africa, HIV testing coverage remains low with up to 54% of adults are unaware of their HIV status, despite substantial efforts to increase HIV testing [4]. The low coverage observed undermines the ability to reach the first "90" target and is a critical barrier to scaling up HIV prevention, care and treatment programmes. Also, late presentation for ART and delay harms treatment outcomes among HIV–positive people in Africa, leading to high mortality, and high transmission of the virus [5, 6].

Existing literature shows that HIV self -testing (HIVST) has the potential to provide an opportunity for individuals who have never tested for HIV to know their HIV status [7]. According to WHO and UNAIDS, HIVST broadly refers to “a process whereby an individual, who is willing to know his/her HIV status collects a specimen, performs the test and interprets the test results in private” [8].

A distinction is made from the mail-in self-testing models of home specimen collection where primarily a person collects a specimen in private and sends it by post to a laboratory for testing and waits for the results [9]. Further, HIVST testing should be regarded as a screening tool only, since they do not provide a definitive diagnosis. A follow-up to a health facility or seeing a health care provider for further confirmatory testing is mandatory after receiving a positive HIVST result [8].

Different from the conventional HIV rapid diagnostics tests (RDTs), which are restricted to health care providers, HIVST consists of approaches for testing products specifically designed and marketed for use by laypersons [10–12]. In response to HIVST recommendations, several African countries have incorporated the HIVST approach in their national HTS policy [11, 13, 14]. Other countries are at different stages in considering including HIVST in policy and practices [11]. Also, results from a five-year (2015–2020), Unitaid-funded HIV Self-testing Africa (STAR) initiative, which supports HIV self-testing in five sub-Saharan countries informed WHO HIVST guidelines [15].

Systematic reviews have been conducted in high- and low and middle -income countries on the acceptability of HIVST [16], performance accuracy among laypersons [17], and the uptake of HIVST [18]. Since the last reviews in 2014, several new studies on HIVST in Africa have been published.

By 2010, new evidence showed that people living with HIV (PLHIV) would benefit more if ART were initiated much earlier. The ART eligibility criteria were revised from a CD4 cell count of 200 to 350 based on the evidence [4]. The changes increased the number of PLHIV enrolled in ART by 1.5 times by the end of 2010 compared to 2008 indicating an increase in expectation and demand for ART [4]. In 2013, new evidence emerged which showed more benefits associated with much earlier initiation of ART and the WHO recommended that the cut–off point for initiation of ART for PLHIV should be a CD4 cell count of 500 [4]. The current guideline suggests that PLHIV should initiate ART irrespective of the CD4 cell count [3]. Linkage to HIV prevention, care and treatment is the ultimate goal for making people in the general population, aware of their HIV status. However, existing evidence suggested that only about 18% of ineligible ART patients at diagnosis remain continuously in care until ART eligibility. Several main reasons for poor retention at pre–ART care documented includes perceived lack of therapeutic benefits compared to cost of attending clinic, risk of losing employment due to frequent clinic visits, cost of transport or fear of visibility as clients of an HIV clinic among asymptomatic patients, and death among those with very low CD 4 counts [19].

According to the WHO definition, social harms, or adverse events refers to any undesirable experience or intended or unintended harm related to HIV testing, including self-testing [8]. Existing evidence so far does not support incidences of social harm directly related to HIVST, however, the WHO guideline underscores the importance of assessing social harm related to HIVST [8].

This systematic review aims to synthesize the evidence on the effects of HIVST among adults in Africa on the uptake of testing, the yield of new HIV–positive diagnoses, linkage to ARV treatment, and the incidence of social harms.

## Methods

This systematic review followed guidance from the Cochrane Collaboration [20] and is reported based on the Preferred Reporting Items for Systematic Reviews and meta-analyses (PRISMA) statement. A review protocol was developed and registered in the PROSPERO International Prospective Register of systematic reviews: (http://www.crd.york.ac.uk/PROSPERO/ PROSPERO registration number: CRD42015023935) [21].

### Eligibility criteria

#### Study designs

Experimental studies including randomized controlled trials (RCTs), before/after studies, and interrupted time-series studies were eligible for inclusion in this review.

#### Participants

The study participants were adults (males and females) from African countries.

#### Intervention and comparisons

HIV self–testing refers to a process whereby an individual, who is willing to know his/her HIV status collects his/her specimen, performs the HIV test and interprets the results in private at home or in other settings. HIVST can be delivered with direct assistance, for example, an in-person demonstration on how to use self-test, or unassisted using manufacturer instructions alone. In this review, we compared the effects of HIV self-testing, either alone, or in addition to the standard HIV testing services (HTS). Standard HIV testing services (HTS) was defined as: routine HTS services provided by trained health care providers at health facilities or HIV testing points, and might involve: (1) provider-administered testing, (2) door-to-door testing, (3) mobile testing, (4) index testing, (5) workplace testing, and (6) client-initiated testing, or some combination of these approaches. We also compared different approaches to HIVST.

#### Outcomes

The primary outcomes were: uptake of HIV testing and the yield of new HIV-positive diagnoses among adults in African countries. The primary outcomes were defined as:

- Uptake: the proportion of participants offered HIV testing who underwent HIV testing [22].
- The yield of new HIV–positive diagnoses: the proportion of participants offered HIV testing who were newly diagnosed as HIV-positive [23, 24].

The secondary outcomes were defined as:

- HIV positivity: the proportion of participants offered HIV testing who was diagnosed with HIV positive [25].
- Linkage to ARV treatment: the proportion of diagnosed HIV-positive participants who were enrolled in ARV treatment at any time after testing.
- CD4 count: the proportion of diagnosed HIV-positive participants who had their CD4 count measured [26].
- Social harm: the number of participants for whom any episode of harm was observed or reported, during or after HIV testing (e.g. intimate partner violence, coercive testing by a partner, or suicide, etc.) [27].

#### Inclusion and exclusion criteria

All experimental studies which included RCTs, before/after studies and interrupted time-series studies, and compared HIVST with the standard of HTS and/or which compared different approaches to HIVST were eligible for inclusion. Editorials, reviews, perspectives, and studies not evaluating self–testing strategies (e.g. home-based but non-self–test) were excluded. Studies, which did not clearly define the type of HIV testing strategies, were also excluded. Abstracts were included if full-texts were not available. Any disagreements in study inclusion/exclusion were resolved by consensus during a meeting between reviewers.

#### Setting

We included studies conducted in any country on the African continent.

### Information sources

#### Electronic databases

A comprehensive search strategy was developed to identify both published and unpublished articles with no language restrictions from January 1998 to December 2019. The start-time period search restriction was used because since 1998 there has been an emergence of the advanced development of rapid HIV diagnostic tests including self–testing [28]. The review searched for related studies in PubMed, The Cochrane Central Register of Controlled Trials (CENTRAL), Pan African Clinical Trials Registry, The Cochrane Database of Systematic Reviews (CDSR), Databases of Abstracts of Reviews of Effectiveness (DARE), Social Sciences Citation Index, Web of Science and African Index Medicus. Also, we searched websites and databases for grey materials (generally refers to no publicly published literature) such as greynet.org, World Health Organization Library Information System (WHOLIS), WHO Global Index Medicus, The Joint United Nations Programme on HIV/AIDS (UNAIDS resource library), Alliance of Health Policy & Systems Research, The World Bank. We also searched for conference abstracts of the following conference databases: International AIDS Conference 1st International symposium on self-testing for HIV, International AIDS Society Conference (IAS), and Conference on Retroviruses and Opportunistic Infections (CROI). Terms for self-testing only were used to search for HIV-related conference abstracts published between 2001–2019 to overcome the limitations of search functions. We also searched the HIV Self-Testing Research and Policy Hub (HIVST.org) for ongoing updates of ongoing studies. The search strategies for electronic databases incorporated medical subject headings (MeSH), free–text terms and comprehensive African search filter that was adapted to suit each database using an applicable controlled vocabulary [29, 30]. We also checked reference lists of included studies for other eligible reports. We also checked reference lists of the included studies and other relevant systematic reviews for further eligible reports using Google Scholar, and Web of Science. Before the publication of this review, we conducted an updated search for HIVST studies in Africa that were published from 1^st^ January 1998 to 31^st^ December 2019.

#### Search strategy

This systematic review used a search strategy described in a previously published protocol and summarized in Additional file 1 [21].

The search was restricted to human subjects. The full search string for PubMed is included as supplementary material S1 File. We sought expert views on the search strategy to assess the search terms proposed for this review.

### Study records

#### Data management

All search results were merged into reference management software EndNote and were all imported into Convidence (https://www.Convidence.org/), and BN and DD performed an automatic check to exclude duplicate entries.

#### Selection process

**Fig 1:**
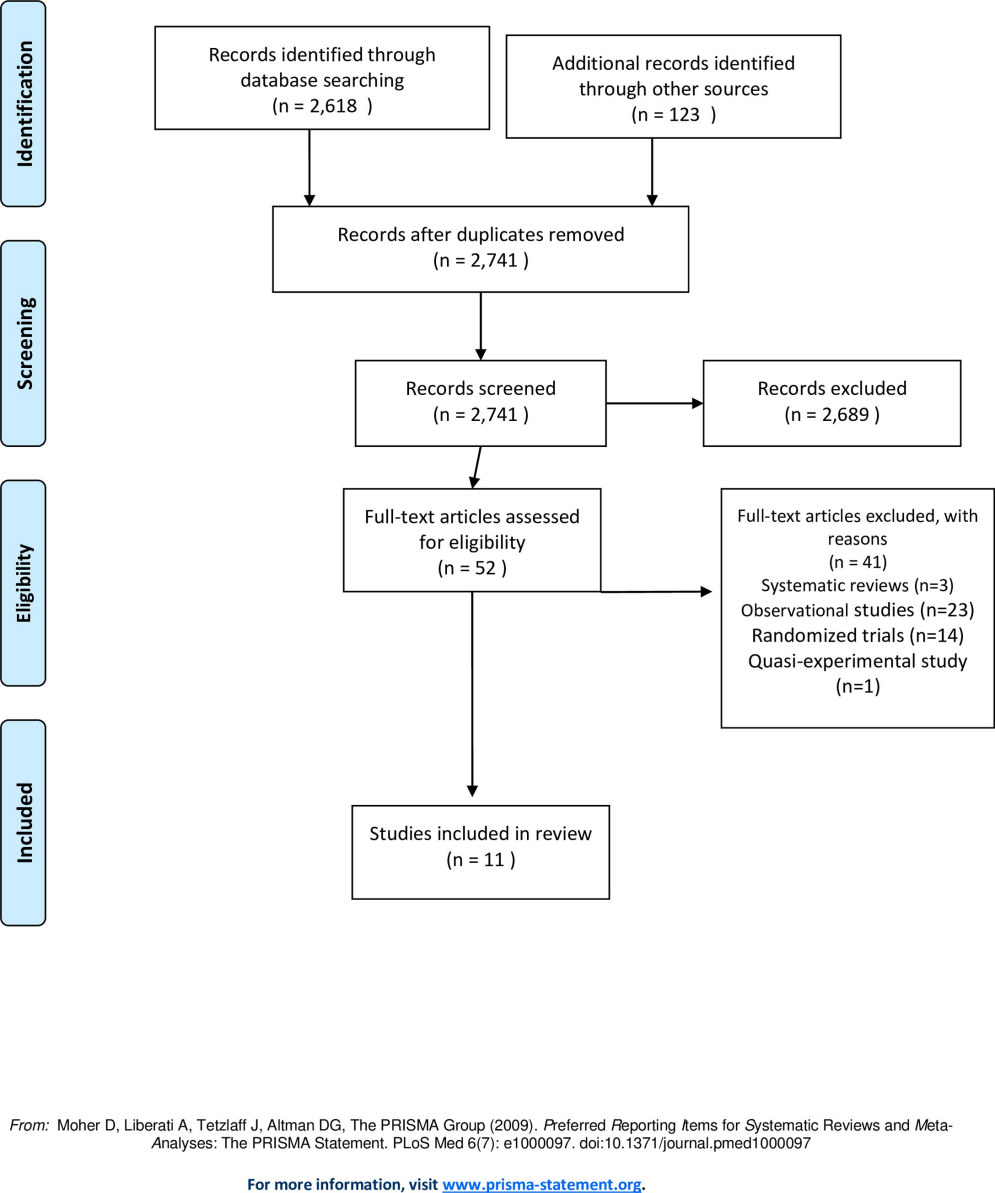
Flow diagram through different phases of the review.

Two reviewers (BN and DD) independently screened the titles to exclude all irrelevant studies and select potentially eligible studies. In this process of abstract screening, the two reviewers included, excluded or classified abstracts as ‘maybe’. The reviewers met to discuss the disagreements, with a consensus to include or exclude. BN then obtained the full text of potentially eligible studies and later BN and DD independently conducted the final study selection for inclusion in the review. The two reviewers provided the reasons for excluding studies at this stage. The final included studies for data collection and the screening process created by Convidence are presented as part of the PRISMA study flow chart shown in Fig 1.

#### Data collection process

Two reviewers, independently extracted data using a pre-designed data extraction form. A third reviewer (LA) was consulted to resolve any differences of opinion between the two reviewers that arose. We conducted a pilot trial of the data extraction form to check its adequacy and made necessary changes.

#### Data items

Extracted data included: study design, setting of the study (e.g. city/ country/or rural/urban or facility-based /community–based), year of study, year of publication–1998 to date, type of HIV self–testing (e.g. supervised or unsupervised), type of comparator (e.g. standard HIV testing services), study population (e.g. general population or key populations), and study outcomes of interest.

#### Assessment of risk of bias in included studies

Two authors (BN and DD) independently assessed the risk of bias (low risk of bias, high risk of bias, or unclear risk of bias) using the Cochrane risk of bias instrument, including an evaluation of random sequence generation and allocation concealment (selection bias), blinding of participants and personnel (performance bias), blinding of outcome assessment (detection bias), incomplete outcome data (attrition bias), selective reporting (reporting bias), and unclear risk of bias (either due to lack of information or uncertainty over the potential bias). We resolved disagreements by consensus. Trials reporting > 20% of missing data were judged at a high risk of attrition bias. In HIV testing studies, it is difficult to bind participants, and/or researchers to the interventions resulting in a high risk of performance bias been made. However this should not infer that the methodological quality of the trials was poor, but the reviewer's acknowledging the inevitable bias related to lack of blinding [31]. The assessments of risk of bias are summarized in Tables 1, 3 and 4 and Fig 6.

**Table 1:** Study and intervention characteristics of eleven RCTs (n = 11).

**First author**	**Study design /Sample characteristics**	**Comparison condition**	**Intervention condition**	**Findings**	**Quality of evidence**
Masters et al. 2016 [35]	Two - arm randomized controlled trial (1:1). A total of 1929 women were screened and 600 pregnant and post-partum women aged between 18 and 39 years in urban & peri-urban, Kenya, presenting at post-partum or antenatal care, with no risk of IPV, and who haactd a male partner with unknown HIV status or known HIV-negative status were enrolled and randomized.	Facility- based HIV testing (n= 303 male partners): Participants received an invitation card plus a voucher inviting their male partners for free HIV testing at study clinic. Women participants also received information on clinical counseling, and legal support on IPV. The invitation cards had messages about the importance of testing (i.e., Standard of care [SOC]).	Unsupervised HIVST (n= 297 male partners): Participants received two free Ora Quick RDT with manufacturer’s instructions to distribute to their male partner or to test as a couple, a demonstration on how to use the kits, clinical counseling on how to encourage their male partners to test, and legal support on IPV.	**Primary outcome:** **Uptake of HIV testing** ^**a**^**:** Participants reported at follow-up the uptake of HIV testing by their male partner 36 weeks after her enrollment. 90.8% Intervention Group (IG) vs. 52 % Control Group (CG) [Risk ratio (RR) =1.76; 95% Confidence Interval (CI): 1.56 to 1.98]. **Uptake of couples HIV testing** ^**b:**^ Participants reported uptake of couples HIV testing 36 weeks after enrollment. Couples testing referred to testing together at the same time. 75.4 % IG vs. 33 % CG [RR=2.27; 95% CI: 1.90 to 2.71]. **HIV positivity** ^**c**^ **:** Participants self-reported that their male partners tested HIV-positive. 4.6 % IG vs. 3.5 % CG [RR=1.31; 95% CI: 0.58 to 2.94]. **Linkage to care** ^**d**^**:** Participants with HIV-positive diagnoses and undergo confirmatory HIV testing. 25% IG vs. 75% CG [RR = 0.33; 95% CI 0.09 to 1.26]. **Secondary outcome:** **Social harm** ^**e:**^ Participants reported whether they experienced physical, emotional, verbal, or sexual violence from their partner after enrollment. 0.34 % (n=1/297) IG vs. 0.33% (n=1/303) CG	**Sequence generation:** Participants were randomized in a 1:1 ratio using computer-generated random numbers and using a balanced block randomization (block size of 20) to HIVST group or a comparison. **Allocation concealment:** Participants were offered sealed randomized envelopes sequentially. **Blinding of participants and personnel:** The study did not describe the blinding process. After randomization it was not possible to blind given the nature of the intervention and the comparison groups. **Blinding of outcome assessment:** Study primarily relied on self-reported of outcomes from women of male partners who self-tested. Tracking number of referral vouchers at study facilities validated confirmatory testing following HIVST reactive results. **Incomplete outcome data:** Attrition rate was 5 % (Follow-up was completed for 95% of study participants (n=570/600). **Selective reporting:** Reported nearly all outcomes except confirmatory HIV testing results, and sexual behaviour and decision-making outcomes. **Other potential source of bias:** None
Gichangi et al. 2018 [34]	Three -arm randomized controlled trial (1:1:1) in 14 antenatal care(ANC) sites in Eastern & Central Kenya. 1,410 pregnant women aged 18 years and older attending their first antenatal clinic visit not at risk of IPV, and 1,333 male partners with unknown or known HIV-negative status. However this review only analyzed the comparison between two of the arms (SOC & HIVST). The third arm was excluded from the analysis; it involved PMTC services. Only 943 from the two arms were included in the analysis.	Facility- based HIV testing (n= 471 male partners): Women participants received standard invitation letter to their male partner for HIV testing alone/ or as a couple at a standard of HIV testing care at clinic (SOC).	Unsupervised HIVST (n= 472 male partners): Women participants’ received an improved invitation letter emphasizing the importance of male HIV testing, and 2 free OraQuick RDT with manufacturer’s instructions, to distribute to their male partner to test for HIV alone/or to test as a couple. Women participants also viewed a 10 min. demonstration on how to use the kits, and interpret the results correctly and were counseled on how to encourage their male partners to test, and handle their male partners in case of a positive result.	**Primary outcome:** **Uptake of couples HIV testing** ^**b**^**:** Women participants reported uptake of couples HIV testing at follow-up by reporting that they had tested together with her partner at the same time. 79. 1 % IG vs. 27 % CG (#1) [RR= 2.92 95% CI: 2.47 to 3.45]. **Linkage to care** ^**d**^**:** 72% of male partners testing at home self-reported to undergo confirmatory test ta the health facility.	**Sequence generation:** Participants were randomly assigned using three different colors: Arm 1 was yellow, arm 2 was green, and arm 3 was blue. Coloured stickers were labeled in accordance to each facility’s sample size, and put in sealed envelopes and mixed up. **Allocation concealment:** After consenting participants were asked to pick a sealed envelope and open. Participants were allocated to respective arms determined by the label colour they have picked. **Blinding of participants and personnel:** Study staffs were not blinded to knowing the study group to which each participant was randomized. Participants knew what they are being offered without being told by the study staff. **Blinding of outcome assessment**: Information on blinding of assessors was not available. **Incomplete outcome data:** The study reported high retention rate for women participants (arm 1= 86%; arm 3 = 84%). Male partner follow-up was also high (arm 1=80%; arm 3=84%). Intent-to-treat analysis was done at 3-months. **Selective reporting:** The study reported findings for both primary and secondary outcomes. **Other potential source of bias:** None noted
Chanda et al., 2017 [36]	Three -arm randomized controlled trial (1:1:1), Peer educators enrolled 965 female sex workers (FSWs)aged 18 years of age or older in 3 Zambian transit towns. However this review only analyzed the comparison between two of the arms (SOC & direct delivery of HIVST). The third arm was excluded from the analysis; it involved distribution of coupons for free collection of OraQuick test kits. Only 596 from the two arms were included in the analysis.	Facility based HIV testing (n= 301 female sex workers): Participants received information about existing HIV testing services from peer-educators, including the locations and working hours, where they could obtain an HIV test.	1.Direct delivery of HIVST kit (n= 295 female sex workers): Participants received 2 Ora Quick RDT HIVST kits. Peer educators distributed 2 HIVST kits during their intervention visits to participants in the community (one at the 1^st^ visit (week 0); a second one at the fourth visit (10th week), with manufacturer’s pictorial and written instructions in English and 3 local languages. Peer-educators demonstrated to participants on how to use the kit, results interpretation, and follow-up care. 2. Distribution of a coupon (n= 329 female sex workers). Participants received a coupon from trained peer-educators for a free OraQuick RDT HIVST kit from a health facility or pharmacy. Peer-educators distributed the 2 coupons at the 1^st^ and 4^th^ intervention visits in the community. The content of the test and instructions were identical to those in the delivery arm.	**Primary outcome:** **Uptake of HIV testing** ^**a**^**:** Participants self-reported uptake of HIV testing at 1 month and 4 month study assessments **At 1 month:** 94.9 % IG (1) vs. 88.5 % CG [RR= 1.07; 95% CI: 0.99 to 1.15]. **♯At 4 months:** 84.1 % IG (1) vs. 75. 1 % CG [RR=1.20; 95% CI: 1.11 to 1.29]. **HIV positivity** ^**c**^ **:** Participants self-reported that their most recent HIV test was positive. **At 1 month:** 16.7 % IG(1) vs. 20. 5 % CG [RR= 0.78; 95% CI: 0.51 to 1.02]. **♯At 4 months:** 25.3 IG(1) vs. 28.2 % CG [RR= 0.91; 95% CI: 0.70 to 1.19]. **Started ART**^**f**^**:** Participants with an HIV –positive test result self-reported that they were taking antiretroviral medicines. **At 1 month:** 22. 5% IG(1) vs. 46.6 % CG [RR= 0.55 ; 95% CI: 0.27 to 1.10]. **♯At 4 months:** 48 % IG(1) vs. 64.3% CG [RR= 0.74; 95% CI: 0.55 to 0.98]. **Secondary outcome:** **Social harm** ^**e:**^ Physical, sexual, or verbal intimate partner violence, unintentional disclosure of HIV status, or self harm (self-reported). 0.6% (n= 2/316) IG(1) vs. 0.6 % (n= 2/329) IG (2). No adverse events was reported in the standard of care arm	**Sequence generation:** The randomization list was computer generated in random blocks of size 3,6, and 9 and stratified by 3 study sites to ensure balance in study arms by site. **Allocation concealment:** Participants were sequentially allocated into one of the three study arms using sealed opaque, sequentially numbered envelopes. **Blinding of participants and personnel:** Due to the nature of the intervention, masking was not possible; however the peer educator’s study arm assignment was concealed until all participants had been enrolled. **Blinding of outcome assessment:** Due to the nature of the intervention, masking was not done. **Incomplete outcome data:** The study reported high retention rates for FSWs. Attrition rate: 1 month= 9% 4 months= 7 % **Selective reporting:** Reported results of all ( 1= primary; 4= secondary) study outcomes **Other potential source of bias:** None detected.
Ortblad et al., 2017 [39]	Three -arm randomized controlled trial (1:1:1), Peer educators enrolled 960 female sex workers aged 18 years of age or older in 5 Kampala divisions, Uganda. However this review only analyzed the comparison between two of the arms (SOC & direct delivery of HIVST). The third arm was excluded from the analysis; it involved distribution of coupons for free collection of OraQuick test kits. Only 600 from the two arms were included in the analysis.	Facility based HIV testing (n= 303 female sex workers): Participants received information about existing HIV testing services, including the locations and working hours, from peer-educators.	1.Direct delivery of HIVST (n= 297 female sex workers): Peer educators distributed 2 HIVST kits during their intervention visits to participants in the community(social networks) one at the 1^st^ visit (week 0); a second one at the fourth visit (10th week), with manufacturer’s pictorial and written instructions in English and Luganda - a local language. Peer-educators demonstrated to participants on how to use the kit, results interpretation, and follow-up care. Participants also received free condoms. 2. Distribution of a coupon (n= 336 female sex workers). Participants received a coupon from the trained peer-educators to collect a free 2 Oral Quick RDT HIVST kit from 10 private health facility or pharmacy in exchange for the coupon. Peer-educators distributed the 2 coupons at the 1^st^ and 4^th^ to the participants during the intervention visits in the community (social networks). The content of the test and instructions were identical to those in the delivery arm.	**Uptake of HIV testing** ^**a**^**:** Participants self-reported uptake of HIV testing at 1 month and 4 month study assessments **At 1 month:** 95.2 % IG (1) vs. 71.5 % CG [RR= 1.33; 95% CI:1.17 to 1.51]. **♯At 4 months:** 99.6 % IG (1) vs. 87.1 % CG [RR= 56.93; 95% CI: 23.86 to 135.85]. **HIV positivity** ^**c**^ **:** Participants self-reported that their most recent HIV test was positive. **At 1 month:** 13.4 % IG (1) vs. 12.3 % CG [RR= 1.05; 95% CI: 0.62 to 1.75]. **♯At 4 months:** 9.1 % IG (1) vs. 14.6 % CG [RR= 0.89; 95% CI: 0.59 to 1.35]. **Started ART**^**f**^**:** Participants with an HIV –positive test result self-reported that they were taking antiretroviral medicines. **At 1 month:** 4.5 % IG (1) vs. 4.3 % CG [RR= 0.99; 95% CI: 0.37 to 2.67]. **♯At 4 months:** 7.3 % IG (1) vs. 8.2 % CG [RR= 0.75; 95% CI: 0.49 to 1.14]. **Secondary outcome:** **Social harm** ^**e:**^ Participants were screened for physical, sexual, or verbal intimate partner violence, unintentional disclosure of HIV status, and self harm at each peer educator intervention visit and during study assessments. 0.7 % (n= 2/296) IG(1) vs. 0.6 % (n= 2/336) IG (2). No adverse events was reported in the standard of care arm	**Sequence generation:** The randomization list was computer generated in random blocks of size 3,6, and 9 and stratified by 5 administrative divisions and evenly recruited peers to ensure balance in study arms by site. **Allocation concealment:** Participants were sequentially allocated into one of the three study arms using opaque, sequentially numbered envelopes. **Blinding of participants and personnel:** Due to the nature of the intervention, masking was not possible; however study personnel, peer educators, and participants were masked to study arm assignment prior to opening the sealed envelopes. **Blinding of outcome assessment:** Due to the nature of the intervention, masking was not done. **Incomplete outcome data:** The study reported high retention rate for women participants. Attrition rate was: at 1 month= 4 %; at 4 months= 8.5 % **Selective reporting:** Reported results of all ( 1 = primary ; 6=secondary) study outcomes. **Other potential source of bias:** None detected
Kelvin et al., 2018 [43]	Two -arm randomized controlled trial (1:1), The trial enrolled 305 male truck drivers aged 18 years of age or older in 2 North Star Alliance roadside wellness clinics in Kenya.	Facility –based HIV testing (n= 155). Participants were offered a PITC administered-blood-based (finger prick)HIV test (i.e., Standard of Care [SOC]). Participants were followed up to 6 months, with standard text reminders at 3 months.	Facility and community-based HIVST (n=150). Participants were offered a Choice between SOC and a self-administered oral rapid HIV test with provider supervision in the clinic. Participants who refused HIV testing at the clinic were offered a test kit for home use with phone-based posttest counseling. Participants were followed-up for 6 months, with an option of picking-up a self-administered OraQuick test kit to use at home, and received a standard text reminder at 3 months.	**Primary outcome:** **Uptake of HIV testing** ^**a**^ **:** Participants who accepted HIV testing in the clinic or a test kit for home use. **At follow-up:** 87.3% IG vs. 72.9% SOC [RR= 1.20; 95% CI: 1.07 to 1.34]**.**	**Sequence generation:** Participants were randomized in a 1:1 ratio stratified on clinic. **Allocation concealment:** Participants were assigned to either the SOC arm or the Choice arm by fieldworker from a sealed envelope with the randomization assignment. **Blinding of participants and personnel:** The study did not describe the blinding of participants. Field workers were not blinded after randomization. **Blinding of outcome assessment:** The study did not describe the blinding of outcome assessment. **Incomplete outcome data:** The study used intent-to-treat analysis. The study reported high retention rate for both SOC arm and the Choice arm. Attrition rate at 6-month follow-up was: 8.4% SOC arm, and 5.3% the Choice arm. **Selective reporting:** The study reported results of all study outcomes. **Other potential source of bias:** None detected
Dovel et al., 2018 [37]	Three-arm randomized controlled trial conducted at 15 health facilities (1:1:1) among 5675 outpatients in Central and Southern Malawi. However this review only analyzed the comparison between two of the arms (SOC & HIVST). The third arm was excluded from the analysis; it involved Optimized PITC services. Only 3918 from the two arms were included in the analysis.	Facility based HIV testing (n=1988). Participants attending outpatient clinic were offered a routine PITC (i.e., SOC). Participants were offered an Optimized PITC (OSOC), including additional training, job aids, and monitoring of PITC strategies with OPD providers and support staff.	Facility based supervised HIVST (n=1930). 3. Participants were offered HIVST, including a demonstration, distribution, and testing in outpatient waiting spaces, private spaces for interpretation, and optional post-test counseling.	**Primary outcome:** **Uptake of HIV testing** ^**a**^ **:** Outpatient clients who accepted SOC or facility –based HIVST testing. **52 % IG vs. 12 % SOC**[RR= 3.97; 95% CI: 3.50 to 4.49] **HIV positivity** ^**c**^ **:** HIVST was associated with a higher absolute number of new positives identified compared to SOC (AOR: 4.1, p= 0.002). **Secondary outcome:** **Social harm** ^**e:**^ No adverse events were reported in the HIVST arm.	**Sequence generation:** Participants were randomized in a 1:1:1 ratio using computer-generated random numbers and using a balanced block. 15 clusters were blocked by facility type, ownership, and region and allocated to either the SOC, OSOC, or HIVST arm. **Allocation concealment:** The study protocol did not describe the allocation concealment process. **Blinding of participants and personnel:** The study did not blind participants or personnel. **Blinding of outcome assessment:** This was a non-blinded study. **Incomplete outcome data:** The study used intent-to-treat analysis. Data on retention rate of participants on follow-up are not available. **Selective reporting:** Only preliminary results for HIV testing and HIV positivity are presented. **Other potential source of bias:** None detected
Indravudh et al., 2018 [44]	Parallel two-arm randomized controlled trial (1:1) including 22 public health clinics and there defined catchment areas in rural Malawi, among 8,000 adults aged 15 years or older.	Facility-based HIV testing (n=2908 ). Participants were offered routine HIV testing while visiting health facilities (i.e., Standard of Care [SOC]).	Community-based supervised HIVST (n= 2581). Community-based distributors (CBDAs) distributed HIVST kits door-to-door. Distributors also provided continuous HIVST access and an option of post-test support, and assisted referral to routine confirmatory testing and HIV care and treatment services.	**Secondary outcome:** **Social harm** ^**e**^**:** Three incidences of social harms were reported out of 79,349 HIVST kits distributed. **Started ART**^**f**^**:** The proportion of ART initiations among 93,640 adults per 1000 clinics populations increased in the HIVST vs. SOC RR* 1.36, 95% CI: 0.95-1.94, p= 0.09). * Adjusted for baseline (pre-intervention) ART initiation.	**Sequence generation:** 22 rural PHC were randomized 1:1 to receive the HIVST intervention or SOC. In the HIVST intervention arm, CBDAs were randomized 1:1 to provide either referral to home-based ART or the usual referrals to the clinic. CBDAs were randomized in blocks of varying sizes from 2 to 12 CBDAs. **Allocation concealment:** The study protocol did not describe the allocation concealment process. **Blinding of participants and personnel:** Given the nature of the study the participants and personnel were not blinded to the allocation status. **Blinding of outcome assessment:** Trial statisticians were un-blinded after completion of data collection. **Incomplete outcome data:** Data on retention rate of participants on follow-up are not available. **Selective reporting:** The study presents preliminary results for HIV testing (i.e., in last 12 months and lifetime), and ART initiations. **Other potential source of bias:** None detected
Floyd et al., 2018 [41]	A HIVST cluster-randomised trial nested within a community-based trial in Zambia. A parent three-armed trial: HPTN 071 (PopART) was delivered in 21 urban and peri-urban study clusters in Zambia (n=12), and South Africa (n=9), with a total population appx: 1 million adults aged 15 years and above- in “annual rounds” from 2013-2017. In 2016, 4 out of 12 intervention communities in Zambia, with 66 (33 HIVST vs. 33 Non-HIVST) zones were included in the cluster-randomised trial of adding oraQuick kits to the standard intervention.	Community-based HIV testing (n= 13,706). Community-HIV-care providers (CHiPs) visited all households, and offered home-based HIV testing with RDT using finger-prick blood, and referrals to routine clinic services, and support for linkage to care (i.e., Standard of care [SOC].	Community –based HIVST (n= 13,267) Community-HIV-care providers, offered self-testing in person, supervised or unsupervised, and secondary distribution to absent partners.	**Primary outcome:** **HIV positivity**^**c**^**:** Participants self-reported that their most recent HIV test was positive. 1.46% IG vs. 1.48 SOC [RR= 0.99,95% CI: 0.81-1.20]. **Linkage to care** ^**d**^**:** Participants with HIV positive diagnoses were referred to local primary health facility for HIV treatment and care. 94% IG vs. 98% SOC [RR= 0.97, 95% CI: 0.93-1.01].	**Sequence generation:** In the HPTN 071 trial-randomization was done in public ceremonies simultaneously in Zambia and South Africa. The 21 clusters were initially divided into 7 matched triplets. **Allocation concealment:** 21 study clusters (7 clusters in each study arm) in the HPTN 071 trial were assigned to 3-study arms using restricted randomization process to ensure overall balance across study arms on cluster size, current ART uptake, and HIV prevalence. **Blinding of participants and personnel:** Unclear risk of bias (blinding of participants and personnel not described in the abstract). **Blinding of outcome assessment:** Unclear risk of bias (blinding of outcome assessors not described in the abstract). **Incomplete outcome data:** The study used ‘time-to-event” analysis and follow-up information. **At 3 months after referral:** 64% SOC vs. 65% HIVST were LTC **Selective reporting:** The study presented preliminary results on HIV positivity and linkage to care. **Other potential source of bias:** None detected
Wanyenze et al. 2019 [40]	Two-armed cluster-randomized trial (1:1) among pregnant women attending Antenatal care (ANC) at three- study sites in Uganda. ANC clinic days were randomized into 120 clusters overall.	Facility based HIV-testing (n= 816). Pregnant women were given health education and encouraged to bring their partners for couple’s HIV test at the facility (SOC).	Supervised facility-based HIVST (n=742). Pregnant women were given health education and delivery of four (4) HIVST kits to their male partners and other adult family members in the household.	**Primary outcome:** **Uptake of HIV testing** ^**a**^ **:** Participants ( either the women/male partner) self-reported the uptake of HIV testing across month 1 and month 3. 76. 7 % IG vs. 37.5 % SOC [RR= 2.05; 95% CI: 1.85 to 2.26]. **Uptake of couples HIV testing** ^**b**^**:** Participants (either the women/male partner) self-reported the uptake of couples HIV testing across month 1 and month 3. 73.3% IG vs. 31% CG [RR= 2.38; 95% CI: 2.10 to 2.70]. **HIV positivity**^**c**^**:** Participants self-reported that their most recent HIV test was positive. 7 % IG vs. 4 % CG [RR= 1.70; 95% CI: 0.87 to 3.24]. **Linkage to care** ^**d**^**:** Participants with an HIV –positive test result sought medical care for their HIV infection across month 1 and month 3 follow-ups. 24 % IG vs. 45. 5 % CG [RR= 0.52; 95% CI: 0.23 to 1.22].	**Sequence generation:** Clinic day clusters were randomized in either SOC arm or HIVST arm using generated random numbers each day of recruitment. All women who were recruited from a particular clinic on a particular day were randomized to the same study arm **Allocation concealment:** Participants were sequentially allocated into one of the two study arms using separate lists of random numbers and sampling frames each day. **Blinding of participants and personnel:** Participants and personnel were blinded. **Blinding of outcome assessment:** The study did not describe on blinding of outcome assessors. **Incomplete outcome data:** All analyses were based on intention-to-treat. The study reported high retention rate for both SOC arm and HIVST arm. Attrition rate: **At 3 months:** 15.7% SOC arm, 17.7 % HIVST arm. **At 6-months:** 5.1% SOC arm, 1.4% HVST arm. **Selective reporting:** The study reported findings for all study outcomes. **Other potential source of bias:** None detected
Choko et al. 2019 [42]	An adaptive multi-armed (1:1:1:1:1:1), 2 stage cluster-randomized trial among pregnant women attending Antenatal care (ANC) at three primary health clinics in urban settings in Malawi. ANC clinic days (i.e., clusters of women attending on a single day) were unit of randomization. 2,349 eligible pregnant women attending an ANC for the first time for their current pregnancy (regardless of trimester), 18 years and older, with a primary male partner unknown to be on ART were enrolled in the trial. However this review only analyzed the comparison between two of the arms (SOC & “ST”) . The other 3 arms were excluded from the analysis. Only 850 from the two arms were included in the analysis.	Facility-based HIV testing (n=408). Women attending ANC were given an invitation letter to deliver to their male partner including information on the importance of testing for HIV, the availability of HIV testing, fast track referral for HIV treatment and/or voluntary medical male circumcision (VMMC) services through a male-friendly clinic (i.e., Standard of care[SOC]).	Facility-based HIVST (n=1941). Women in all 5-intervention groups received the SOC letter and clinic assesses and 2 prequalified oral HIVST kits to deliver to their male partners. **# IG # 1(n=442):** Offered SOC letter and 2 self-test kits (“ST”). **IG# 2 (n=380):** Offered SOC letter, 2 self-test kits and additional conditional fixed cash financial incentive of $ 3 (“ST + $3”) when male partners link into a male-friendly clinic and receive HIV care or HIV prevention services. **IG # 3 (n=512):** Offered SOC letter, 2 self-test kits and additional conditional fixed cash financial incentive of $ 10 (“ST+$10) when male partners link into a male-friendly clinic and receive HIV care or HIV prevention services. . **IG # 4 (n= 155):** Offered a 10% chance of winning $ 30 (“ST + lottery) when male partners link into a male-friendly clinic and receive HIV care or HIV prevention services. . **IG # 5 (n= 452):** Offered an additional phone call to the male partner on the day the women were enrolled, and repeated 5 days for a male partner who has not come to the clinic (“ ST+ reminder”).	**Primary outcome:** **Uptake of HIV testing** ^**a:**^ Women participants reported their male partners undergoing HIV testing within 28 days. 92.1 % “ST” vs. 17.4% SOC [RR= 5.29; 95% CI: 4.28 to 6.55]. **HIV positivity**^**c**^**:** Male partners with a newly confirmed HIV positive results. 90.9 % “ST” vs. 100 % SOC [RR= 2.42; 95% CI: 0.71 to 8.27]. **Linkage to care** ^**d**^**:** Male partners referred after confirmed HIV positive results and started ART. 24 % IG vs. 45. 5 % CG [RR= 0.52; 95% CI: 0.23 to 1.22]. **Secondary outcomes:** **Social harm** ^**e**^**:** No severe adverse events were reported.	**Sequence generation:** Clinic days where randomised in blocks of different sizes to any of the six trial arms in stage 1 of the trial. All three-clinic days available in one day where pre-identified before the randomization such that the arm recruiting at a particular clinic on particular day will be known within randomization sequence. **Allocation concealment:** The ANC clinic days were sequentially allocated using computer-generated random numbers by independent statistician. **Blinding of participants and personnel:** Participants and personnel were blinded. **Blinding of outcome assessment:** The study did not describe the blinding of outcome assessors. **Incomplete outcome data:** All analyses were based on intention-to-treat. The denominator was the number of women who were eligible and take into account the clustered design. The study reported high retention rate for both SOC and “ST” arm. Attrition rate: **At 4 weeks follow-up post women enrollment:** 19.57% SOC arm, 17.6% “ST” arm. **Selective reporting:** The study reported findings for all 5 (1=primary; 4=secondary) outcomes. **Other potential source of bias:** None detected
MacPherson et al. 2014 [25]	Two-arm cluster randomized controlled trial (1:1). 16,660 adults aged 16 years and above recruited from 14 clusters in urban Blantyre Malawi. All 14 clusters were restricted randomized into 2 groups: HIVST with optional home initiation of HIV care services or HIVST with facility-based HIV care services only.	Unsupervised home-based HIV ST with facility-based standard HIV care services (n= 8,466): Adult participants’ who requested HIVST received free Oral Quick test kits. Participants who reported HIV positive results could self-refer or referred by the counselor to study clinics for confirmatory HIV testing, and initiation of ART at 1 of the 3 study facilities. ART initiation was ascertained by interviewing all adults who initiated ART at any of the 3 study clinics.	Unsupervised home-based HIVST with optional home initiation of HIV care services (n=8,194): Adult participants’ who requested HIVST received free Oral Quick RDT from the house of volunteer counselors to perform HIVST, and pre-testing information, counseling, and demonstration on how to use the kits. Participants returned the used kit in person to the counselor in a sealed envelope after self-testing. Participants had an option to report or not to report their self-test results to the counselor. Counselors organized home visits by study nurses for participants reporting a positive HIV self-test result who requested home initiation of HIV care. An ART initiation was ascertained by recording all adults who initiated ART at home.	**Secondary outcomes:** **HIV positivity**^**c**^**:** Participants self-reported that their most recent HIV test was positive. 6 % IG vs. 3.3 % CG **[**RR= 1.86, 95 % CI: 1.16 to 2.98**].** **Linkage to care** ^**d**^**:** Participants with an HIV –positive test result self-reported that they have sought medical care for their HIV infection. (i)**CD 4 counts**^**g**^**:** 72.5 % IG vs. 51 % CG [RR= 0.70, 95 % CI: 0.54 to 0.91]. (ii)**Started ART**^**f**^**:** Participants with an HIV –positive test result self-reported that they were taking antiretroviral medicines. 2.2 % IG vs. 0.7 % CG [RR=2.94, 95% CI: 2.10 to 4.12]*.	**Sequence generation:** Reported that 14 clusters were randomized in a 1:1 ratio( 7 clusters into intervention group; 7 clusters into control group). **Allocation concealment:** Used colored balls from an opaque bag held above eye level to select the distribution of clusters and allocation. **Blinding of participants and personnel:** Reported blinding of investigators but not the counselors, or study participants, or outcome assessors. **Blinding of outcome assessment:** Due to the nature of the study masking was not done. **Incomplete outcome data:** All participants completed the trial, attrition rate was: 28.7 % in IG, and 23.8 % in CG from ART, no treatment withdrawals, no trial group changes, and no major adverse events reported. **Selective reporting:** The study reported results of all outcomes (1 primary; 4 secondary). **Other potential source of bias:** None detected.

IG = intervention group; CG = control group; IPV = intimate partner violence; PMCT = Prevention-of-mother-to-child-transmission

^**a**^ Proportion of participants offered HIV testing who underwent HIV testing

^**b**^ Proportion of participants who undergo HIV testing with their partners

^**c**^Proportion of participants with a reactive self-test that received confirmatory testing and diagnosed HIV positive over those who accepted HIV testing

^d^ Proportion of participants who have received confirmatory testing and diagnosed HIV positive self-reporting taking ART medicines over those with an HIV-positive results

^**e**^ Proportion of self-reported instances of intimate partner violence, self-harm, or coercion to test for HIV

^**f**^ Proportion of participants with an HIV-positive test result self-reporting whether they had sought medical care for their HIV infection

^**g**^ Based on WHO criteria (CD4 ≤ 350 cells μl)

^**h**^ Proportion of all resident adults who initiate ART during the first 6 months of the home-based HIV-testing intervention.

*the denominator is all study participants, not HIV positive participants; **♯** data used in meta-analysis.

#### Investigation of heterogeneity

The I^2^ statistics were used to evaluate the heterogeneity of results for each outcome, across trials. We interpreted values of 25, 50, and 75% as low, medium, and high heterogeneity respectively. The observed I^2^ statistics relies on the magnitude and direction of effects, and the strength of evidence for heterogeneity. Although not a measure of absolute heterogeneity, the I^2^ statistics describe the percentage of variability in the point estimates that is due to heterogeneity rather than sampling error [32].

#### Assessment of reporting biases

The review employed strategies to search and include relevant unpublished studies to reduce publication bias. The strategies included searching the gray literature, including conference proceedings (e.g., International AIDS Conference), and prospective trial registration database to over-come lime-lag bias. To investigate publication bias, if there were ≥ 10 trials included in an analysis, we planned to use a funnel plot to explore the possibility of small study effects-a tendency for smaller studies to report larger beneficial effects. However, this review did not use a funnel plot test because of fewer trials (< 10 trials) were included in an analysis, as the power of tests is too low to distinguish chance from publication bias [33].

#### Data analysis

Where data from ≥ 2 trials were available, pooled intervention effect estimates are expressed as risk ratios with corresponding 95% confidence intervals (CI) for each outcome included in the study. The review used the fixed effects models and random-effects models for analysis in the Cochrane Review Manager. Pooled estimates were calculated using random-effects models were high heterogeneity was found among studies. The random-effects model approximates the extent of the heterogeneity and allocates a greater variability to the estimate of overall effect to account for the heterogeneity. In the result section we present forest plots of random-effects models, and forest plots of fixed effects models as Supporting file S2 Fig [33].

#### Sensitivity analysis

Sensitivity analyses were performed on outcomes where an effect of excluding outlier studies and those with high overall bias risk might affect the overall estimate. Visual inspection of forest plots was used to identify trials, which were outliers, and the I^2^ was re-evaluated without the outliers. We also investigated the influence of a single study on the overall estimate by excluding 1 study in each turn [32].

## Results

### Description of included studies

#### Search results

A total of 2,617 citations were identified through electronic search and other sources, which after screening resulted in eleven trials met the review eligibility criteria (Fig 1). There are ongoing trials conducted by The Self-Testing (STAR) Initiative in Malawi (NCT02718274), Zambia (NCT02793804), and Kenya (NCT03135067).

Eleven trials [25, 34–44] conducted between 2011 and 2019, and including 59,119 participants met the eligibility criteria for inclusion in the review (Fig 1 Study flow diagram).

Eight of the trials were reported in full-text articles [25, 34–36, 38, 40, 42, 43, 45], and three were reported in conference abstracts [37, 41, 44]. Of the 11 trials, three were from Kenya [34, 35, 38, 43], two from Zambia, [36, 41], two from Uganda, [39, 40] and four from Malawi [25, 37, 42, 44]. Four trials were conducted in facility-based [34, 37, 40, 42], two were community-based [41, 44], and five were both facility-based and community-based [25, 35, 38, 41, 43, 44]. Five trials used direct assisted HIVST [25, 35, 37, 40, 44], two used unassisted HIVST [34, 39], and one trial used both [43]. Four trials aimed to increase the uptake of HIV testing among male partners of pregnant women attending antenatal clinics or women attending postnatal clinics after the birth of a child, [34, 35, 40, 42], two trials among female sex workers [36, 39], two trials among adult patients attending health facilities [37, 44], one trial among male truck drivers[38, 43], and three trials were conducted among adults aged 16 years and older from the general population [25, 41, 44]. Across all 11 trials, none reported on the yield of new HIV-positive diagnoses, (one of our primary outcomes), and instead reported HIV positivity (n = 7).

Ten of the trials compared HIVST to standard HTS [34–37, 40–45], and one trial compared the optional home-based confirmatory testing and initiation of antiretroviral therapy (ART) to HIVST accompanied with referral to facility-based HIV care services [25]. All 11 trials used free oral fluid-based HIVST, followed by confirmatory HIV rapid diagnostic tests, and none used finger stick/ or whole blood-based RDTs or both oral and blood specimens.

#### Excluded studies

There were 41 excluded studies, out of which 23 were observational studies/ or other study designs[27, 46–67], three systematic reviews [16–18], 14 randomized trials [68–81], and one quasi-experimental study [82] (see Table 2).

**Table 2:** Characteristics of excluded studies (n = 41).

Study	Reason for exclusion
Wawer et al. 1998 [68]	Intervention ineligible and comparison. STD treatment was used as an intervention, and anthelminthic and iron-folate was used as control in this RCT.
Leon et al. 2010 [69]	Ineligible intervention. Provider-initiated (opt-out) HIV counseling and testing was used in this controlled trial.
Luganda et al. 2010 [70]	Ineligible intervention and comparison. Home-based and clinics-based HIV counseling and testing were used as an intervention and comparison in this RCT.
Choko et al. 2011 [46]	A cross-sectional feasibility study to determine the uptake and accuracy of oral kits for HIV self-testing in high HIV prevalence setting in Blantyre, Malawi.
Sweat et al. 2011 [72]	Ineligible intervention and comparison. Home-based and clinics-based HIV counseling and testing were used as an intervention and comparison in this RCT.
Wanyenze et al. 2011 [73]	Ineligible intervention. Provider-initiated (opt-out) HIV counseling and testing was used in this RCT.
Baisley et al. 2012 [74]	Ineligible intervention. Voluntary Counseling and testing was used as an intervention in this trial in Northwest Tanzania.
Pant Pai et al. 2013 [66]	Supervised and Unsupervised Self-Testing for HIV in High- and Low-Risk Populations: A Systematic Review.
Krause et al. 2013 [17]	Acceptability of HIV self-testing: a systematic literature review.
Suthar et al. 2013 [18]	A Systematic Review and Meta-Analysis of Community-Based Approaches.
Pant Pai et al. 2013 [66]	A cross sectional pilot feasibility study among health care workers provided with an unsupervised self-testing strategy for HIV in South Africa.
Doherty et al. 2013 [74]	Ineligible intervention. Home-based HIV counseling and testing was used as an intervention in this RCT.
Fylkesnes et al. 2013 [75]	Ineligible intervention. Home-based HIV counseling and testing was used as an intervention in this RCT.
Low et al. 2013 [76]	Ineligible intervention. Home-based HIV counseling and testing was used as an intervention in this RCT.
Kebede et al. 2013 [47]	Ineligible study design. A cross-sectional study.
Wanyenze et al. 2013 [77]	Ineligible intervention. Abbreviated HIV counseling and testing was used in this a factorial RCT.
Asiimwe et al. 2014 [78]	A randomized implementation trial among fisher folk in 3 communities at high risk of HIV infection in Uganda. The trial did not address the outcomes of this review.
Kalibala et al. 2014 [48]	A cross-sectional study to determine factors associated with acceptability of HIV self-testing among health care workers in Kenya.
Coates et al. 2014 [79]	Ineligible intervention. Community based-Voluntary counseling and testing was used as an intervention in this RCT.
Labhardt et al. 2014 [80]	Ineligible intervention and comparison. Home-based HIV counseling and testing and mobile clinics were used as an intervention and comparison in this RCT.
Kumwenda et al. 2014 [49]	A cross-sectional study to determine factors shaping initial decision-making to self-test amongst cohabiting couples in urban Blantyre, Malawi.
Peck et al. 2014 [50]	A usability study of test prototypes in unsupervised HIV self- testing in Kenya, Malawi, and South Africa.
Brown et al. 2015 [51]	A study conducted among HIV stakeholders to explore public opinion and perspectives on HIV self-testing in Nigeria.
Choko et al. 2015 [27]	A community-based prospective study to assess the uptake, accuracy, safety, and linkage into care over two years of promoting annual self-testing for HIV in Blantyre, Malawi.
Kurth et al. 2016 [52]	A cross-sectional study to assess the accuracy and acceptability of oral fluid HIV self-testing in a general adult population in Kenya.
Mavedzenge et al. 2016 [53]	A cross-sectional study to determine acceptability, feasibility, and preference of HIV self-testing in Zimbabwe.
Thirumurthy et al. 2016 [54]	A cohort study to assess an approach of providing multiple self-tests to women at high risk of HIV acquisition to promote partner HIV testing and to facilitate safer sexual decision-making in Kenya.
Perez et al. 2016 [55]	A cross-sectional study on supervised oral HIV Self-testing in accurate in rural Kwazulu, Natal, South Africa.
Mugo et al. 2017 [56]	A feasibility study to determine uptake and acceptability of oral HIV self-testing among community pharmacy clients in Kenya.
Agot et al. 2018 [57]	Conference abstract; data included in the review.
Sibanda et al. 2017 [81]	Ineligible intervention. A non-monetary incentive was used as an intervention in this cluster-randomized trial.
Chipungu et al. 2017 [58]	Ineligible study design. A cross-sectional study.
Zanolini et al. 2017 [59]	Ineligible study design. A discrete choice experiment study.
Tonen-Wolyec et al. 2018 [60]	Ineligible study design. A multicenter cross-sectional study.
Achia et al. 2018 [82]	Conference abstract; full study not available.
Bwana et al. 2018 [61]	Ineligible study design. A cross-sectional study.
George et al. 2018 [62]	Ineligible study design. A cost analysis study.
Ahmed et al. 2018 [63]	Ineligible study design. A cost analysis study.
Sibanda et al. 2018 [64]	Ineligible study design. A discrete choice experiment study.
Inwani et al. 2018 [65]	Conference abstract; ineligible study design.
Lebina et al. 2019 [67]	Ineligible study design. A prospective study.

### HIVST compared with standard HIV testing services

#### Uptake of HIV testing

**Fig 2:**
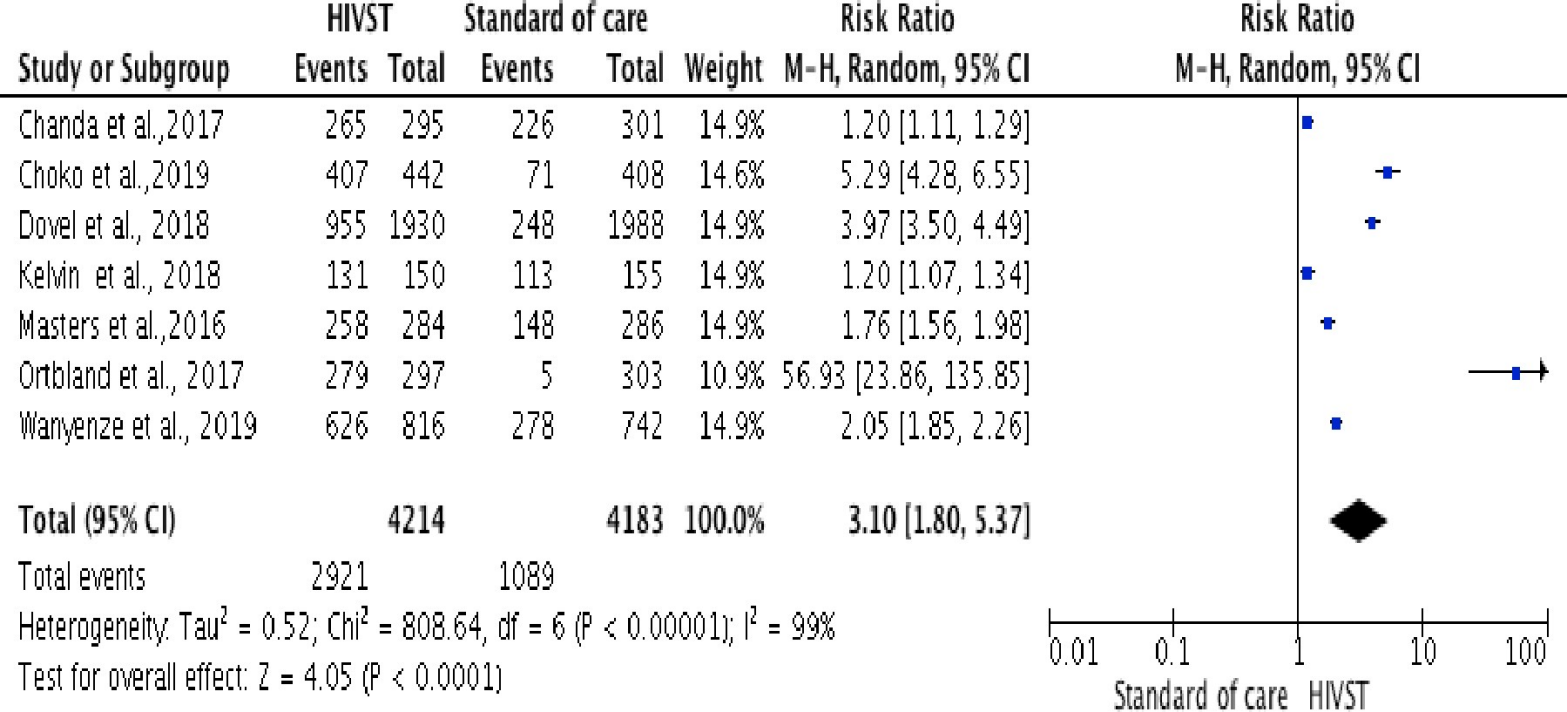
Forest plot: Comparison of HIVST versus standard of HIV testing services; outcome: Uptake of HIV testing. The RR and horizontal lines represent the 95% CI in random-effect model.

Three trials in Kenya, Malawi, and Uganda [35, 40, 42], recruited women (n = 5450), with no risk of intimate partner violence, and with their male partners of unknown HIV status or known HIV-negative status, at antenatal and postnatal care and invited them to either distribute HIV self-tests to their male partners (intervention arm) or an invitation card and referral vouchers for free HIV testing, here assumed to be standard HIV testing services (comparison arm). Two trials conducted in Zambia and Uganda [36, 39] recruited adult female sex workers (n = 1,925), who reported the exchange of any vaginal, anal, or oral sex for money, goods, or other items of value and invited them to either direct delivery of HIV self-tests, or coupons for free collection of HIV self-tests (intervention arms) or referral for standard HIV testing services (comparison arm). One trial in Kenya [43] recruited male truck drivers (n = 305) and offered a self-administered HIVST under the supervision/ or un-supervised self-administered HIVST at home (intervention arms) or routine PITC HIV testing, here assumed to be standard HIV testing services (comparison arm). One trial in Malawi [37] recruited adult patients attending the out-patient department (n = 3918) and offered self-administered HIVST (intervention arms), or routine PITC HIV testing, here assumed to be standard HIV testing services (comparison arm). A meta-analysis showed an increase in the uptake of HIV testing in the HIVST arm compared with the standard HTS arm (RR = 3.10, 95% CI: 1.80 to 5.37; Tau^2^ = 0.52; Chi^2^ = 808.64; df = 6 (p < 0.00001; I^2^ = 99% in random-effects models)). The I^2^ of 99% is the proportion of the variation in observed effects due to variation of the true effects (Fig 2).

**Fig 3:**
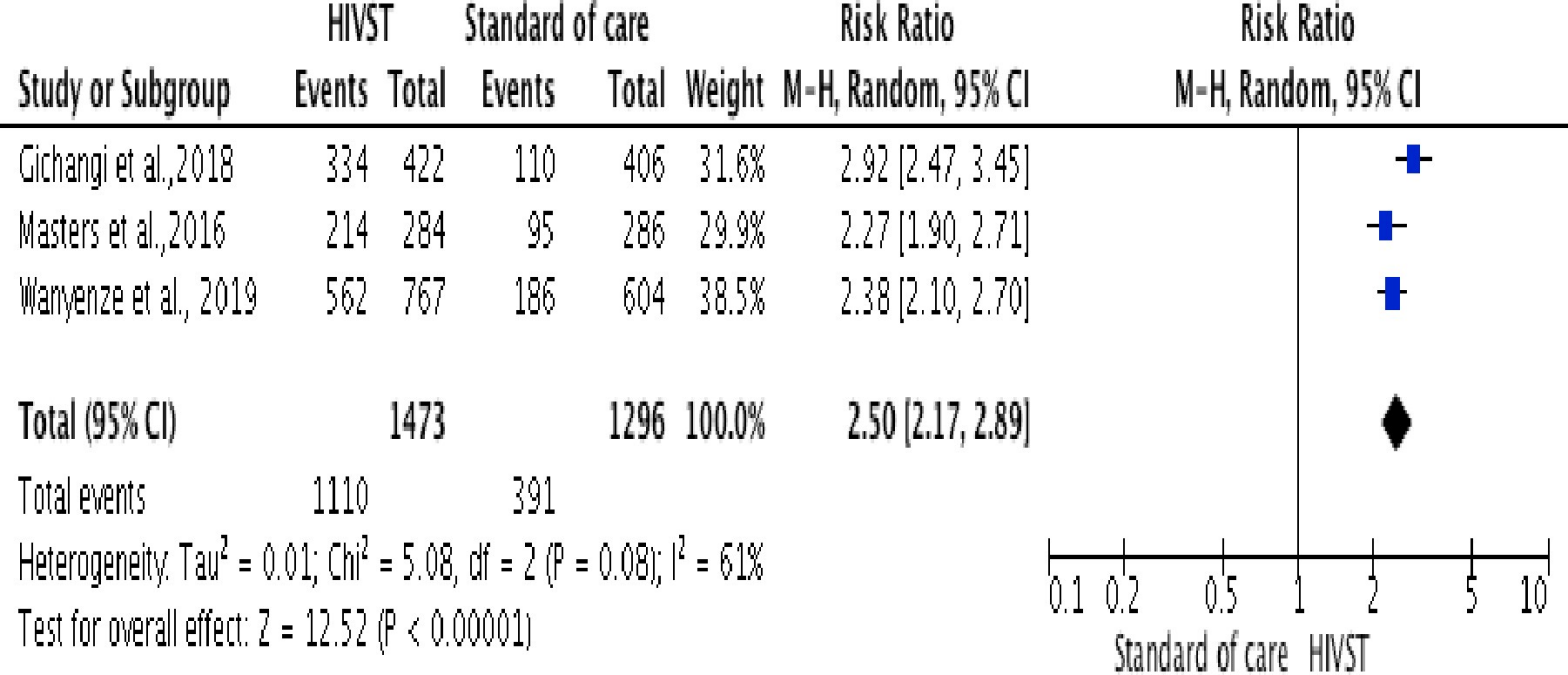
Forest plot: Comparison of HIVST versus standard of HIV testing services; outcome: Uptake of couple’s HIV testing. The RR and horizontal lines represent the 95% CI in random-effect model.

A meta-analysis including three trials [34, 35, 40], among pregnant women (n = 8,397), showed an increase in the uptake of couples HIV testing in the HIVST arm compared to standard HTS (RR = 2.50, 95% CI: 2.17 to 2.89; Tau^2^ = 0.01; Chi^2^ = 5.08; df = 1 (P = 0.08; I^2^ = 61% in random-effects models)). Couples HIV testing referred to the participant and her male partner testing for HIV together at the same time. The I^2^ of 61% is the proportion of the variation in observed effects due to the variation of the true effects (Fig 3).

#### HIV positivity

**Fig 4:**
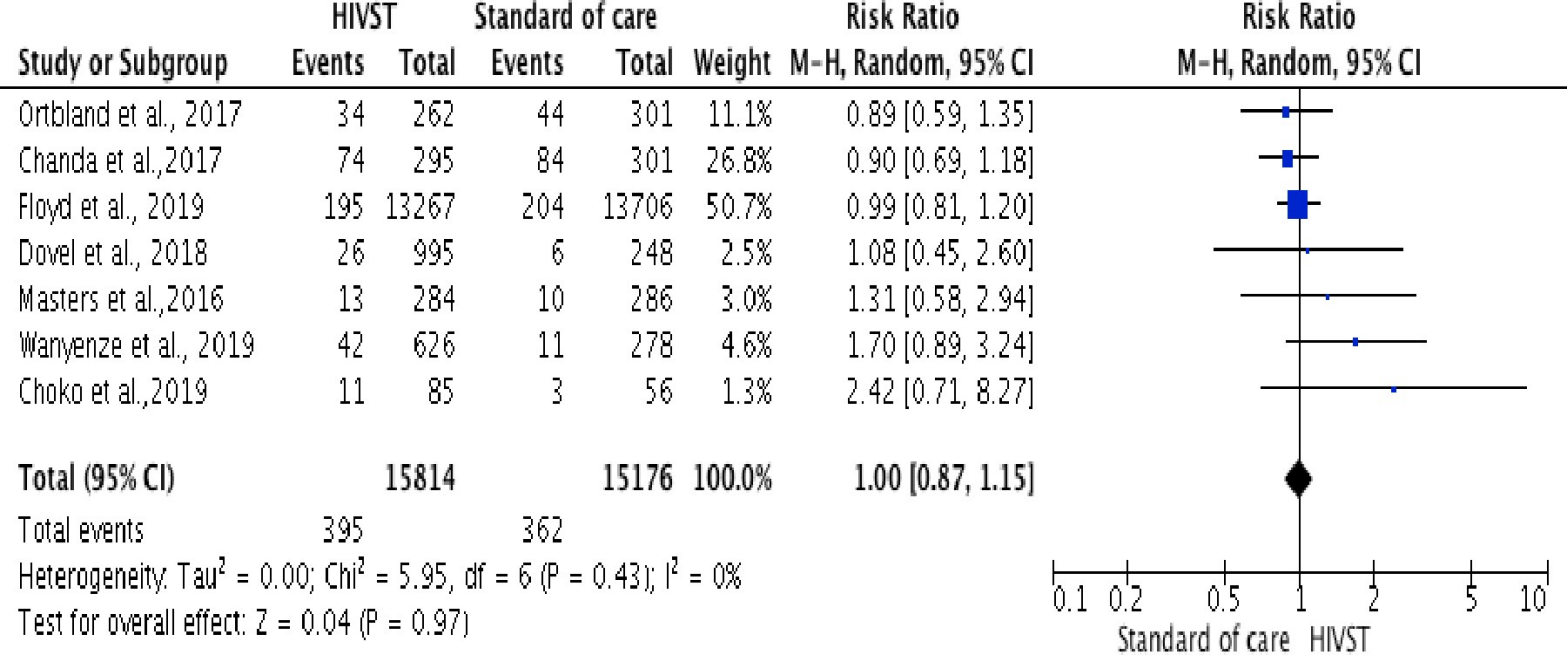
Forest plot: Comparison of HIVST versus standard of HIV testing services; outcome: HIV positivity. The RR and horizontal lines represent the 95% CI in random-effect model.

Seven trials [35–37, 40–42, 45], assessed HIV positivity among male partners of pregnant women (n = 3), female sex workers following HIV testing (n = 2), adult patients attending health facilities (n = 1), and adults in the general population (n = 1). HIV positivity rate was determined as the proportion of participants diagnosed HIV-positive over those who accepted HIV testing. A meta-analysis showed no statistically significant difference in reporting HIV positivity among male partners of pregnant women, and female sex workers from HIVST compared to standard HTS (RR = 1.00, 95% CI: 0.87 to 1.15; Tau^2^ = 0.00; Chi^2^ = 5.95; df = 6 (p < 0.43; I^2^ = 0% in random-effects models)). The I^2^ of 0% is the proportion of the variation in observed effects due to variation of the true effects (Fig 4).

#### Linkage to care and antiretroviral treatment

**Fig 5:**
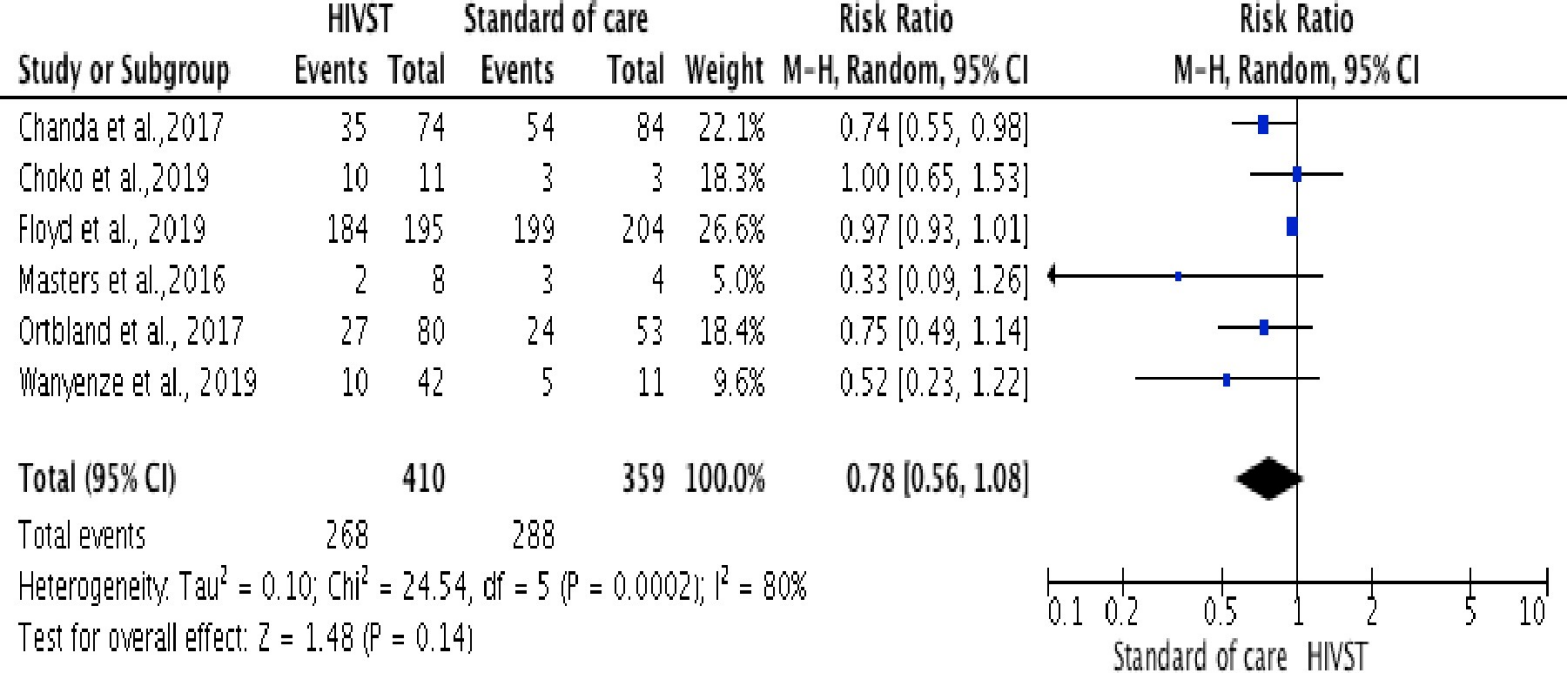
Forest plot: Comparison of HIVST versus standard of HIV testing services; outcome: Linkage to HIV care and ART. The RR and horizontal lines represent the 95% CI in random-effect model.

Six trials, [35, 36, 40–42, 45], reported on linkage to care and ART among participants who were diagnosed HIV positive (n =). A meta-analysis showed that participants from HIVST are less likely to seek HIV–related care and initiating ART compared to participants from HTS (RR = 0.78, 95% CI: 0.56 to 1.08; Tau^2^ = 0.10; Chi^2^ = 24.54; df = 5 (p < 0.0002; I^2^ = 80% in random-effects models)). The I^2^ of 80% is the proportion of the variation in observed effects due to variation of the true effects (Fig 5).

#### Social harms

Six trials assessed social harms, following HIVST or standard HTS among adult participants. The trial in Kenya [35] reported a single incidence of harm in each group between two HIV-negative participants in the HIVST group, and the control group. In both groups, the reported harm was verbal and/ or physical intimate partner violence. The trial in Zambia [36] reported four incidences of harm, with three participants in the direct delivery of HIVST intervention arm, and one participant in the coupon intervention arm, reporting physical violence following primary partner learning of their HIVST use. None incidence of harm was reported in the standard of care arm. The trial in Uganda [39], reported four incidences of harm, with one participant in the direct delivery of HIVST intervention arm, and one participant in the coupon intervention arm, reporting verbal abuse following primary partner learning of their HIVST use/or mental distress following a positive HIV self-test result. Two participants in the standard of care arm reported verbal abuse after the disclosure of their HIV status. Two trials among male partners of pregnant women and patients attending health facilities in Malawi reported no social harms after HIVST [37, 42]. Another trial in Malawi among the the general population of rural communities reported three incidences of social harm [44].

#### Sensitivity analysis

Exclusion of 5 studies from a meta-analysis reporting uptake of HIV testing in HIVST arm compared with the standard of HTS arm altered the overall estimate and heterogeneity (RR = 1.20, 95% CI: 1.12–1.27; P = 0.99, I^2^ = 0%). Exclusion of studies from meta-analyses reporting uptake of couples HIV testing, HIV positivity, and linkage to care and ART did not alter the existing heterogeneity.

### HIVST with optional home-initiation of HIV care compared to HIVST with facility-based HIV care

One RCT in Malawi (n = 16,660) compared different approaches to HIVST [25].

#### HIV positivity

The trial reported HIV positivity among participants following HIV testing. HIV positivity rate was determined as the proportion of participants diagnosed HIV-positive over those who accepted HIV testing. There was a significant difference in reporting a positive test result between the home and facility groups (RR: 1.86; 95% CI: 1.16 to 2.98; P = .010) to HIVST accompanied with referral to facility-based HIV care services.

#### Tested for CD4 counts

The study compared the receipt of CD4 count test results among newly diagnosed HIV-positive individuals. In the arm with the offer of optional home-based confirmatory testing and initiation of ART group, 72. 5% (n = 79/109) of newly diagnosed HIV positive individuals received CD4 count results compared to 51% (n = 23/63) in the facility group (RR = 0.70, 95% CI: 0.54 to 0.91; P = .007).

#### Linkage to care and antiretroviral treatment

The trial reported ART initiation and retention in care. In the arm with the offer of optional home-based confirmatory testing and initiation of ART group, initiation of ART was 2.2% (n = 181/8,194) compared to 0.7% (n = 63/8,466) in the control arm (RR, 2.94; 95% CI: 2.10 to 4.12; P < .0001). After adjusting for reported household mortality at baseline, the effect of availability of home ART care was (ARR, 2.44; 95% CI: 1.61 to 3.68; P < .001).

#### Assessment of risk of bias in included studies

**Fig 6:**
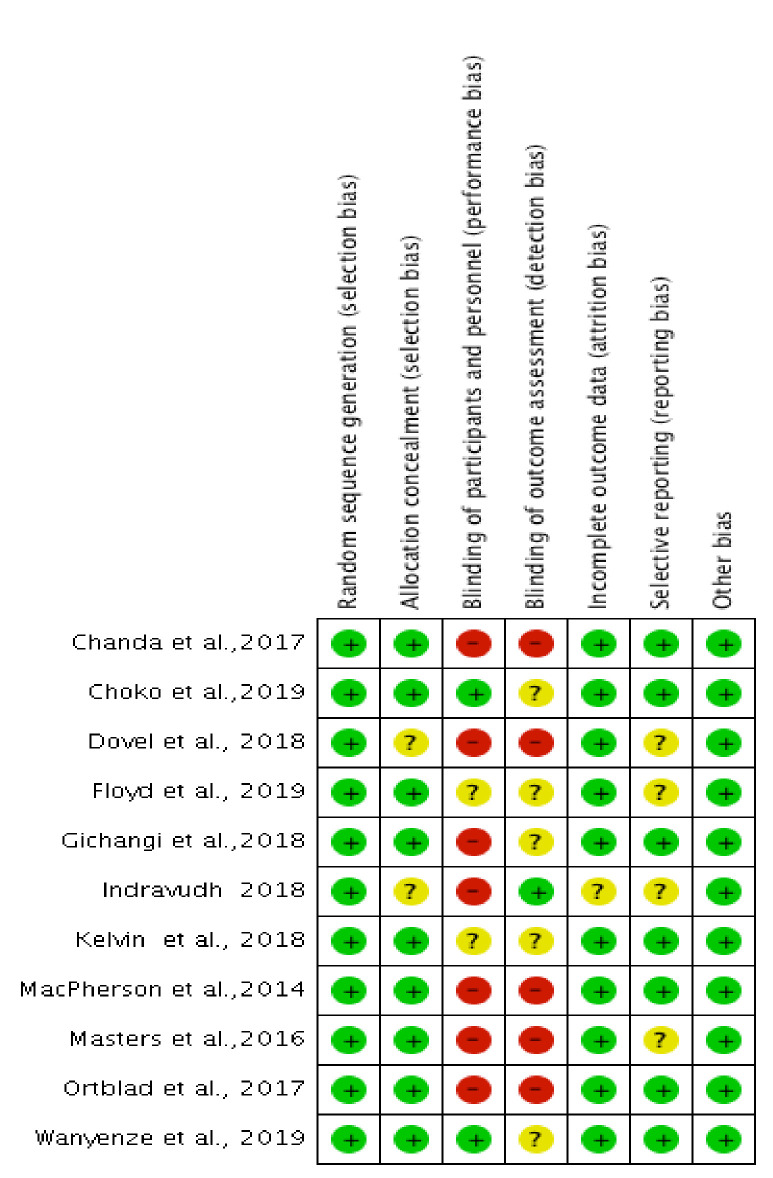
Risk of bias summary: Review authors' judgments about each risk of bias item for each included study.

The risk of bias in the included studies was assessed as described above. The assessments of risk of bias in included studies are presented in the risk of bias graph below (Fig 6), while additional details are included in Tables 1, 3 and 4.

**Table 3:** HIV self-testing compared to the standard of HIV testing services for adults.

Patient or population: Adults aged 16 years and above.					
Settings: Rural and urban settings in Africa.					
Intervention: HIV self-testing					
Comparison: Standard of HIV testing services					
Outcomes	Illustrative comparative risks* (95% CI)		Relative effect	No of Participants	Quality of the evidence
			(95% CI)	(studies)	(GRADE)
	Assumed risk	Corresponding risk			
	Standard of care	HIV self-testing			
Uptake of HIV testing (direct delivery of HIVST versus standard of care)					
	371 per 1000	901 per 1000	RR 3.10	8397	⊕⊕⊕⊝^1^^,^^2^^,^^3^^,^^4^
		(422 to 1000)	(1.80 to 5.37)	(7 studies)	moderate
Uptake of couple HIV testing					
	302 per 1000	549 per 1000	RR 2.50	2769	⊕⊕⊕⊝^5^^,^^6^
		(477 to 631)	(2.17 to 2.89)	(3 studies)	moderate
HIV positivity (direct delivery of HIVST versus standard of care)					
	411 per 1000	386 per 1000	RR 1.00	30990	⊕⊕⊝⊝^7^^,^^8^
		(312 to 477)	(0.87 to 1.15)	(7 studies)	low
Started ART (direct delivery of HIVST versus standard of care)					
	574 per 1000	414 per 1000	RR 0.78	303	⊕⊕⊝⊝^7^^,^^8^
		(322 to 513)	(0.56 to 1.08)	(6 studies)	low

*The basis for the assumed risk (e.g. the median control group risk across studies) is provided in footnotes. The corresponding risk (and its 95% confidence interval) is based on the assumed risk in the comparison group and the relative effect of the intervention (and its 95% CI).

CI: Confidence interval; RR: Risk ratio.

GRADE Working Group grades of evidence

High quality: Further research is very unlikely to change our confidence in the estimate of effect.

Moderate quality: Further research is likely to have an important impact on our confidence in the estimate of effect and may change the estimate.

Low quality: Further research is very likely to have an important impact on our confidence in the estimate of effect and is likely to change the estimate.

Very low quality: We are very uncertain about the estimate.

^1^ Risk of bias: Downgraded the quality of evidence by 1 level for risk of bias. Allocation concealment was judged”unclear” in 1 trial, and the risk of bias cannot be excluded.

^2^ Inconsistency: Downgrading for inconsistency was not done, despite a high statistical heterogeneity. However, the estimates of effects were all in the same direction.

^3^ Indirectness: Did not downgrade for indirectness as the results are directly related to the review question.

^4^ Imprecision: Did not downgrade for imprecision, despite a few numbers of events and a wide confidence interval around the estimate of the effect in one trial.

^5^ Risk of bias: Downgraded the quality of evidence by 1 level for risk of bias. Blinding of outcome assessment was judged “unclear”, and the risk of bias cannot be excluded.

^6^ Inconsistency: Downgrading for inconsistency was not done, despite observed heterogeneity. However, the estimates of effects were all in the same direction.

^7^ Risk of bias: Downgraded the quality of evidence by 1 level because the outcomes were self-reports and performance could not be excluded.

^8^ Imprecision: Downgraded the quality of evidence by 1 level because of the few numbers of events in both groups, and overlapping of confidence interval around the estimate of effect.

**Table 4:** HIVST with optional home initiation of HIV care compared to HIVST with facility-based HIV care services for adults.

Patient or population: Adults aged 16 years and above recruited from the general population.					
Settings: Urban settings in Africa					
Intervention: HIVST with optional home-based HIV care services					
Comparison: HIVST with facility-based HIV care services					
Outcomes	Illustrative comparative risks* (95% CI)		Relative effect	No of Participants	Quality of the evidence
			(95% CI)	(studies)	(GRADE)
	Assumed risk	Corresponding risk			
	HIVST with facility-based HIV care	HIVST with home-based HIV care			
HIV positivity					
	3 per 100	6 per 100	RR 1.86	16,660	⊕⊕⊕⊝^1^^,^^2^
		(5 to 7)	(1.16 to 2.98)	(1 study)	moderate
Tested for CD 4 count					
	51 per 100	36 per 100	RR 0.70	16,660	⊕⊕⊕⊝^1^^,^^2^
		(27 to 47)	(0.54 to 0.91)	(1 study)	moderate
Started ART (direct delivery of HIVST versus standard of care)					
	1 per 100	2 per 100	RR 2.94	16,660	⊕⊕⊕⊝^1^^,^^2^
		(2 to 3)	(2.10 to 4.12)	(1 study)	moderate

*The basis for the assumed risk (e.g. the median control group risk across studies) is provided in footnotes. The corresponding risk (and its 95% confidence interval) is based on the assumed risk in the comparison group and the relative effect of the intervention (and its 95% CI).

CI: Confidence interval; RR: Risk ratio.

GRADE Working Group grades of evidence

High quality: Further research is very unlikely to change our confidence in the estimate of effect.

Moderate quality: Further research is likely to have an important impact on our confidence in the estimate of effect and may change the estimate.

Low quality: Further research is very likely to have an important impact on our confidence in the estimate of effect and is likely to change the estimate.

Very low quality: We are very uncertain about the estimate.

^1^ Single study.

^2^ Risk of bias: Downgraded the quality of evidence by 1 level for risk of bias. Concerns on the blinding of participants, counselors, and outcome assessors, and the risk of bias cannot be excluded.

#### Assessment of overall quality of the evidence

We used the Grading of Recommendations Assessment, Development and Evaluation (GRADE) approach to assess the quality of evidence and generate absolute estimates of effect for the outcomes as described in the GRADE profiler (GRADEpro) software [31, 83]. The GRADE methodology defines the quality of evidence for each outcome as ‘ the extent to which one can be confident that an estimate of effect or association is close to the quantity of specific interest” [20]. The quality rating across studies has four levels: high, moderate, low or very low. RCTs are initially categorized as providing high-quality evidence with the option of downgrading the quality. Quality of evidence can be downgraded based on limitations in design, indirectness of evidence, unexplained heterogeneity or inconsistency of results, imprecision of results or high probability of publication bias [20]. We did not downgrade based on lack of blinding alone due to difficulties of blinding participants, and or research personnel in HIV testing intervention trials. Downgrading was done based on the risk of bias when a lack of blinding was accompanied by additional high risks of bias (e.g., selection bias and incomplete outcome reporting). We summarized the quality of evidence for the studies separately for each outcome in the GRADE Summary of Findings Tables [31].

#### The overall quality of evidence

The overall quality of evidence for the 10 trials [34–37, 39–44] was moderate. The quality of evidence was downgraded because of the risk of bias, imprecision, and inconsistency (Table 3).

The overall quality of the evidence for the cluster RCT [25] was moderate. The quality of evidence was downgraded because of the risk of bias (Table 4).

## Discussion

### Summary of main results

The objective of this systematic review was to synthesize the evidence on the effects of HIVST among adults in Africa on the uptake of testing, the yield of new HIV–positive diagnoses, linkage to ARV treatment, and the incidence of social harms. This review included eleven trials conducted in Malawi, Kenya, Zambia, and Uganda.

Two models of HIV testing were assessed in the included studies of this review. The first model included ten trials comparing HIVST versus standard HTS, and the second model included one trial comparing different approaches to HIVST. In the first model, 10 RCTs in Kenya, Zambia, and Uganda found a positive effect on the uptake of HIVST compared to standard HTS. Similarly, in three trials in Kenya and Uganda reported a positive effect on the uptake of couples' HIVST compared to standard HTS.

Seven trials reported no statistically significant difference in reporting HIV positivity among participants from HIVST compared to standard HTS. Also, six trials reported no statistically significant difference in linkage to care and ART among HIV positive participants from standard HTS compared with those from HIVST. Finally, ten incidences of any kind of social harm were reported following HIVST and standard HTS from 6 RCTs in Kenya, Zambia, and Uganda.

In the second model, one trial in Malawi reported a statistically significant difference, showing participants who received HIVST and an optional home-based confirmatory testing and initiation of ART were more likely to report HIV positive test results than those in the control group. Further, the trial showed an increased proportion of newly diagnosed HIV positive participants in the homegroup received CD 4 count tests than those in the control group. Finally, the trial reported a statistically significant difference showing that participants who reported HIV-positive test results from home-based HIVST with optional home-based confirmatory testing and initiation of ART were more likely to initiate ART care compared with their counterparts from the home-based HIVST with facility-based HIV care.

### Summary of evidence: HIVST versus standard of HIV testing services

#### Uptake of HIVST

Seven trials were included in the meta-analysis, and showed a moderate quality of evidence, for the uptake of HIV testing compared to standard HTS. Furthermore, two RCTs were included in the meta-analysis also showed the moderate quality of evidence for uptake of couple HIV testing. Although the results of the meta-analysis report that HIVST can double the uptake of HIV testing compared to standard HTS, there is some uncertainty because of heterogeneity in effect size between the studies. Besides, the evidence was limited to male partners of pregnant and post-partum women and adult female sex workers.

#### HIV positivity

Seven trials included in this review with moderate evidence showed no difference between HIVST compared to standard HTS in reporting HIV- positivity. The quality of evidence was downgraded for the risk of bias, detection bias, selection bias, and imprecision. None of the trials reported the yield of new HIV positive diagnoses. Yield and HIV positivity are important for designing appropriate and sustainable HIV testing interventions because it determines the number of tests needed to diagnose a new case of HIV, and has cost and resource implications [84]. However, all trials included in this review, none reported on the yield of new HIV positive diagnoses, underscoring the need to assess this important indicator in future studies, particularly in high HIV prevalence settings.

#### Linkage to HIV care

Six trials included in this review with moderate evidence showed poor linkage to HIV care following confirmatory testing between HIV positive participants in the HIVST group compared to those in the standard HTS group.

#### Social harms

One trial from Kenya compared the distribution of HIVST kits by pregnant women to their male partners with standard HTS, and 2 trials from Zambia and Uganda, compared the delivery of HIVST kits with standard HTS among adult female sex workers and assessed IPV resulting from HIVST. The trial reported a total of 10 incidences of any form of social harm related to HIV self-testing reported by different adult populations, such as pregnant women, and female sex workers. It is possible that self-testing does alter IPV experiences, but is currently not accurately measured by the extant literature. This finding of reported rare cases of IPV related to HIVST is consistent with several studies assessing harm across all forms of HIV testing services [27, 85, 86]. However, it is important to introduce HIVST with caution among vulnerable populations (e.g. female sex workers (FSW)) [8, 54].

### Summary of evidence: Comparison of different approaches to HIVST

#### HIV positivity

One trial from Malawi with moderate-quality evidence, assessed different approaches to HIVST and, observed that participants from home-based HIVST with optional home-based confirmatory testing and initiation of ART were significantly more likely to report a positive result than participants from home-based HIVST with facility-based HIV care [25].

#### Tested for CD 4 count

The same trial showed a high proportion of newly diagnosed HIV-positive participants, from the intervention arm compared to participants form the control arm tested for CD4 counts [25]. The finding supports the evidence that a high proportion of CD4 counts could be achieved outside the health facilities when offered together with HIV test results, and may facilitate immediate treatment for improved health and to decrease the probability of further HIV transmissions [18, 27].

#### Started ART

Additionally, the same trial in Malawi reported an increased proportion of participants who reported HIV-positive results from the intervention arm compared to the control arm. However, this RCT reported the loss to follow-up 6 months after the home-initiation of ART compared with the facility-initiation of ART. ART initiators in the homegroup had higher rates of loss from ART compared with their counterparts in the facility group.

#### Overall completeness of the evidence and applicability of evidence

The observed results on increased uptake concur with results from other systematic reviews [16–18, 87, 88] and observational studies from Kenya [56], Lesotho [89], Malawi [27, 46], and Zimbabwe [90], which reported similar increases in the uptake of HIV testing through HIVST. For instance, the systematic reviews reported an uptake of HIVST ranging from 50% to 96% in other parts of the world [16–18] and 20% to 74% in the key populations [18, 88]. Also, a two-year prospective study in Malawi reported s consistently high uptake of community-based HIVST among adolescents aged 16–19 years of age; young people aged 16–29 years of age, women, and men [27].

Although HIVST primarily focuses on an individual, two trials in this review were conducted in Kenya where women distributed HIVST kits to their male partners. The trials also reported an increased uptake of the couple’s testing from HIVST compared with the approach of giving male partners invitation letters or vouchers for free HTS. These results suggest that men may be more likely to prefer HIVST than standard HTS [91]. This observation underscores the importance of getting men to utilize HTS because it is well documented that men compared with women are known to underutilize HTS and present late for care ending up with worse outcomes on treatment [5, 6].

Observational studies among key populations, including men who have sex with men (MSM) [88], people who inject drugs (PWIDs) [92], and female sex workers [93], revealed high uptakes of HIV testing following the offer of HIVST. It is well documented that to achieve universal knowledge of HIV status, it is imperative to have innovative interventions such as HIVST to increase access to HIV testing. This review confirms that HIVST is an important strategy to improve awareness of HIV status among adults in SSA, and provides additional HIV testing options to other approaches such as VCT, PITC, school-based, and work-place testing [8, 92].

Earlier HIV diagnosis supports timely access to ART, with several beneficial consequences, such as improved life expectancy, reduced HIV transmission, decreased stigma related to HIV testing, and the provision of HIV prevention interventions [87]. These results are consistent with results from observational studies among the general populations in SSA, which reported HIV-positivity ranging from 3 to 14% [27, 53, 54, 90], and from 1 to 30% among key populations [54, 93]. As coverage of HIV testing increases, the proportion of HIV-positive tests and new HIV diagnoses will be likely to decrease, and hence calls for more focused HIV testing methods to continue to achieve the same or higher levels of HIV-positivity [8, 92].

This review identified gaps in the evidence on linkage to care and ART. Linkages to further HIV testing and HIV prevention, treatment, and care services are a critical component in the HIV testing cascade. This is more important in HIVST because it requires linkage to confirmatory testing, particularly among those who report a positive HIV test result [87]. The most probable explanation to the observed low level of linkage could be due to a few diagnosed HIV-positives, under-reporting and the possibility that some men were aware of their HIV-positive status and already in care. These findings suggest that innovative follow-up strategies post HIVST such as the use of mobile phones or short message services, or facilitated HIV care assessments and ART initiation interventions could be put into place to encourage linkage [8, 27, 87, 92].

HIV testing experts recognize the importance and complexity of monitoring, reporting, evaluating and mitigating social harms related to HIV testing. The rare cases of social harm related to HIVST reported in this review, help to alleviate a major concern of HTS experts, regarding IPV related to HIVST. The findings may suggest that HIVST does not directly influence the risk of IPV, but the risks are largely context-specific, including the settings (e.g. urban/rural) and the relationship dynamics of couples and partners. To emphasize this critical concern, the WHO recommends that HIV testing programmes consider context-specific strategies to implement HIV testing approaches, including HIVST [8, 91]. Furthermore, fear of status disclosure or stigma, the possibility of false-positive diagnoses, lack of confirmatory HIV testing, and insufficient quality control procedures highlight the need to address legal and human rights issues related to HIVST [8, 85, 87].

#### Quality of the evidence

According to the GRADE system, well-conducted RCTs provide high-quality evidence, while observational studies provide low-quality evidence. This review included only trial data, and we found the quality of evidence reported from trials for the HIVST versus standard HTS was generally moderate. The model for comparison of different approaches for HIVST, including one high-quality trial provided moderate quality of evidence.

#### Potential biases in the review process

We used a broad search strategy to capture as many studies as possible. We limited our search to those studies conducted in Africa from 1998 to date to improve comparability. A random-effects model was used to pool data. Despite using a broad search strategy, we were able to capture eleven trials (8 full test studies, and 3 abstracts) conducted across four countries in SSA. Further, there are limited studies from other parts of Africa, which may limit the generalizability of the review findings.

We, therefore, cannot exclude the possibility that of the dispersion presented, what proportion is due to variance in true effects rather than sampling error [32]. Publication bias was not formally assessed because of the limited number of trials for each outcome, since analytical methods such as funnel plots and funnel plots asymmetry tests, were not appropriate [20].

#### Agreements and disagreements with other studies or reviews

To the authors’ knowledge, this is the first systematic review evaluating the effects of HIVST on the uptake of HIV testing, linkage to care and ART, and social harms among adults in Africa. Previous reviews focused on HIVST strategies, the acceptability of HIVST, and different community-based HIV testing approaches globally. The findings of this review provide important information on the potential of HIVST as an option for HTS among adults in Africa. A key finding of our review is that the uptake of HIVST is promising among different adult populations in both rural and urban settings in Africa, particularly in SSA. Importantly, it is the observation of high participation by male partners in couples' HIV testing through HIVST. However, more data are needed on the yield of new HIV positive diagnoses, particularly from countries with high HIV prevalence in SSA in terms of the diverse range of context and settings.

Further research among key populations such as MSM, PWIDs, and vulnerable populations such as adolescents, and factors associated with their participation in HIVST, is important to inform policy and practice. In addition, further key areas for research includes: the effectiveness of HIVST in detecting previously undiagnosed HIV infection, or number of repeat non-testers, linkage to care following a reactive self HIV test result or a confirmatory positive test, retention of care among those identified HIV-positive, linkage to prevention services among participants with negative results (e.g. male circumcision), and social harms from HIVST, which are important to help guide policy on HIVST in Africa. Interventions to facilitate a timely linkage to care are ongoing in Zimbabwe (PACTR20160700171788), and Malawi (ISRCTN18421340), with preliminary findings reporting significant benefits on linkage to VMMC and ART using financial and non-financial incentives.

#### Implication for research

This review has reported moderate-quality evidence for the uptake of HIVST, suggesting that self-testing has the potential to provide an innovative strategy to increase uptake of HIV testing and increase awareness of HIV status among undiagnosed adults in Africa, particularly in SSA. However, we also reported no statistically significant difference in reporting HIV positivity between participants from HIVST and HTS, indicating a need for further research particularly in high HIV prevalence settings in SSA. This review recommends that high-quality evidence from trials would provide valuable insight into whether HIVST as an additional HIV testing option could facilitate early detection, early linkage to HIV care, treatment, and prevention. Additional trial findings will provide data on HIVST as an invaluable tool for the health authorities of African governments to increase access to HIV care, treatment, and prevention to achieve the "95-95-95" global target by the year 2030.

## Supporting information

S1 Checklist PRISMA 2009 checklist. (PDF)

S1 File Search strategies. (PDF)

S1 Fig Forest plot: Comparison of HIVST versus standard of HIV testing services; outcome: Uptake of HIV testing. The RR and horizontal lines represent the 95% CI in fixed-effect model.
(DOCX)

S2 Fig Forest plot: Comparison of HIVST versus standard of HIV testing services; outcome: Uptake of couple’s HIV testing. The RR and horizontal lines represent the 95% CI in fixed-effect model.
(DOCX)

S3 Fig Forest plot: Comparison of HIVST versus standard of HIV testing services; outcome: HIV positivity. The RR and horizontal lines represent the 95% CI in fixed-effect model.
(DOCX)

S4 Fig Forest plot: Comparison of HIVST versus standard of HIV testing services; outcome: Linkage to HIV care and ART. The RR and horizontal lines represent the 95% CI in fixed-effect model.
(DOCX)

S5 Fig Forest plot: Comparison of HIVST versus standard of HIV testing services; outcome: Uptake of HIV testing. The RR and horizontal lines represent the 95% CI in fixed-effect model.
(DOCX)

S6 Fig Forest plot: Comparison of HIVST versus standard of HIV testing services; outcome: Uptake of couple’s HIV testing. The RR and horizontal lines represent the 95% CI in fixed-effect model.
(DOCX)

S7 Fig Forest plot: Comparison of HIVST versus standard of HIV testing services; outcome: HIV positivity. The RR and horizontal lines represent the 95% CI in fixed-effect model.
(DOCX)

S8 Fig Forest plot: Comparison of HIVST versus standard of HIV testing services; outcome: Linkage to HIV care and ART. The RR and horizontal lines represent the 95% CI in fixed-effect model.
(DOCX)
