# Supplementary material for: The effects of HIV self-testing on the uptake of HIV testing, linkage to antiretroviral treatment and social harms among adults in Africa: A systematic review and meta-analysis
Source: PLoS One. 2021 Jan 27;16(1):e0245498. doi: 10.1371/journal.pone.0245498 (PMC7840047; doi:10.1371/journal.pone.0245498)
Supplement: S1 File — (PDF) [file pone.0245498.s002.pdf]

Search strategy in PubMed		
**Search**	**Query**	**Items**
#14	Search # 3 AND # 11 AND # 12 AND # 13 AND (with publication date from Jan 1998 to Dec 2019)	197
#13	(''Africa''[MeSH] OR Africa*[tw] OR Algeria[tw] OR Angola[tw] OR Benin[tw] OR Botswana[tw] OR ''Burkina Faso''[tw] OR Burundi[tw] OR Cameroon[tw] OR ''Canary Islands''[tw] OR ''Cape Verde''[tw] OR ''Central African Repub- lic''[tw] OR Chad[tw] OR Comoros[tw] OR Congo[tw] OR ''Democratic Republic of Congo''[tw] OR Djibouti[tw] OR Egypt[tw] OR ''Equatorial Guinea''[tw] OR Eritrea[tw] OR Ethiopia[tw] OR Gabon[tw] OR Gambia[tw] OR Ghana[tw] OR Guinea[tw] OR ''Guinea Bissau''[tw] OR ''Ivory Coast''[tw] OR ''Cote d'Ivoire''[tw] OR Jama- hiriya[tw] OR Jamahiryia[tw] OR Kenya[tw] OR Lesotho[tw] OR Liberia[tw] OR Libya[tw] OR Li- bia[tw] OR Madagascar[tw] OR Malawi[tw] OR Mali[tw] OR Mauritania[tw] OR Mauritius[tw] OR Mayote[tw] OR Morocco[tw] OR Mozambique[tw] OR Mocambique[tw] OR Namibia[tw] OR Niger[tw] OR Nigeria[tw] OR Principe[tw] OR Reunion[tw] OR Rwanda[tw] OR ''Sao Tome''[tw] OR Senegal[tw] OR Seychelles[tw] OR ''Sierra Leone''[tw] OR Somalia[tw] OR ''South Afri- ca''[tw] OR ''St Helena''[tw] OR Sudan[tw] OR Swaziland[tw] OR Tanzania[tw] OR Togo[tw] OR Tunisia[tw] OR Uganda[tw] OR ''Western Sahar- a''[tw] OR Zaire[tw] OR Zambia[tw] OR Zimba- bwe[tw] OR ''Central Africa''[tw] OR ''Central African''[tw] OR ''West Africa''[tw] OR ''West African''[tw] OR ''Western Africa''[tw] OR ''Wes- tern African''[tw] OR ''East Africa''[tw] OR ''East African''[tw] OR ''Eastern Africa''[tw] OR ''East- ern African''[tw] OR ''North Africa''[tw] OR ''North African''[tw] OR ''Northern Africa''[tw] OR ''Northern African''[tw] OR ''South Afri- can''[tw] OR ''Southern Africa''[tw] OR ''South- ern African''[tw] OR ''sub Saharan Africa''[tw] OR ''sub Saharan African''[tw] OR ''subSaharan Africa''[tw] OR ''subSaharan African''[tw]) NOT (''guinea pig''[tw] OR ''guinea pigs''[tw] OR ''aspergillus niger''[tw])	108,240
#12	(randomized controlled trial [pt] OR controlled clinical trial [pt] OR randomized [tiab] OR placebo [tiab] OR drug therapy [sh] OR randomly [tiab] OR trial [tiab] OR groups [tiab]) AND quasi-experimental[MeSH Terms];after[All Fields] OR (pre[All Fields] AND post[All Fields] AND ("research design"[MeSH Terms] OR test[Text Word]))) OR controlled[All Fields]) OR ("interrupted time series analysis"[MeSH Terms] OR interrupted time series[Text Word])) OR CBA[All Fields] " research design"[MeSH Terms] OR test[Text word]	254,366
#11	Search #4 AND # 5 AND # 6 AND # 7 AND # 8 AND # 9 AND # 10	12,928
#10	"self injurious behaviour"[All Fields] OR "self-injurious behavior"[MeSH Terms] OR ("self-injurious"[All Fields] AND "behavior"[All Fields]) OR "self-injurious behavior"[All Fields] OR ("self"[All Fields] AND "injurious"[All Fields] AND "behavior"[All Fields]) OR "self injurious behavior"[All Fields]) OR ("self-injurious behavior"[MeSH Terms] OR ("self-injurious"[All Fields] AND "behavior"[All Fields]) OR "self-injurious behavior"[All Fields] OR ("self"[All Fields] AND "harm"[All Fields]) OR "self harm"[All Fields])) OR ("suicide"[MeSH Terms] OR "suicide"[All Fields])) OR ("suicide, attempted"[MeSH Terms] OR ("suicide"[All Fields] AND "attempted"[All Fields]) OR "attempted suicide"[All Fields] OR ("attempted"[All Fields] AND "suicide"[All Fields]))) OR ("self destructive behaviour"[All Fields] OR "self-injurious behavior"[MeSH Terms] OR ("self-injurious"[All Fields] AND "behavior"[All Fields]) OR "self-injurious behavior"[All Fields] OR ("self"[All Fields] AND "destructive"[All Fields] AND "behavior"[All Fields]) OR "self destructive behavior"[All Fields])) OR ("suicidal ideation"[MeSH Terms] OR ("suicidal"[All Fields] AND "ideation"[All Fields]) OR "suicidal ideation"[All Fields])) OR (suicidal[All Fields] AND ("behaviour"[All Fields] OR "behavior"[MeSH Terms] OR "behavior"[All Fields]))) OR ("intimate partner violence"[MeSH Terms] OR ("intimate"[All Fields] AND "partner"[All Fields] AND "violence"[All Fields]) OR "intimate partner violence"[All Fields])) OR ("spouse abuse"[MeSH Terms] OR ("spouse"[All Fields] AND "abuse"[All Fields]) OR "spouse abuse"[All Fields])	16,347
#9	"antigens"[MeSH Terms] OR antigens[Text Word]) OR (antigen[All Fields] OR antigen/cea[All Fields] OR antigen/factor[All Fields] OR antigen's[All Fields] OR antigenetic[All Fields] OR antigenic[All Fields] OR antigenically[All Fields] OR antigenicity[All Fields] OR antigenotoxic[All Fields] OR antigens[All Fields] OR antigens/immunology[All Fields] OR antigensynthesis[All Fields] OR antigentically[All Fields])) OR CD4[All Fields]) OR ("t-lymphocytes"[MeSH Terms] OR T-Cell[Text Word])) OR receptors[All Fields]) OR ("cd4 antigens"[MeSH Terms] OR CD4 receptors[Text Word])) OR ("t-lymphocytes"[MeSH Terms] OR T Cell[Text Word])) OR T[All Fields]	484,169
#8	(antiretroviral therapy, highly active[MeSH] OR anti-retroviral agents[MeSH] OR antiviral agents[MeSH:NoExp] OR ((anti[tiab]) AND (hiv[tiab])) OR antiretroviral*[tiab] OR ((anti[tiab]) AND (retroviral*[tiab])) OR HAART[tiab] OR ((anti[tiab]) AND (acquired immunodeficiency[tiab])) OR ((anti[tiab]) AND (acquired immuno-deficiency[tiab])) OR ((anti[tiab]) AND (acquired immune-	49,408

	deficiency[tiab])) OR ((anti[tiab]) AND (acquired immun*[tiab]) AND (deficiency[tiab])))	
#7	"hiv"[MeSH Terms] OR "hiv"[All Fields]) AND ("genetic linkage"[MeSH Terms] OR ("genetic"[All Fields] AND "linkage"[All Fields]) OR "genetic linkage"[All Fields] OR "linkage"[All Fields])) OR (("genetic linkage"[MeSH Terms] OR ("genetic"[All Fields] AND "linkage"[All Fields]) OR "genetic linkage"[All Fields] OR "linkage"[All Fields]) AND ("hiv"[MeSH Terms] OR "hiv"[All Fields]) AND care[All Fields])) OR (("genetic linkage"[MeSH Terms] OR ("genetic"[All Fields] AND "linkage"[All Fields]) OR "genetic linkage"[All Fields] OR "linkage"[All Fields]) AND care[All Fields])) OR (link[All Fields] AND care[All Fields])) OR (link[All Fields] AND ("hiv"[MeSH Terms] OR "hiv"[All Fields]) AND care[All Fields])) OR (("hiv"[MeSH Terms] OR "hiv"[All Fields]) AND link[All Fields])	**7,336**
#6	(Yield[All Fields] OR (("hiv"[MeSH Terms] OR "hiv"[All Fields]) AND ("epidemiology"[Subheading] OR "epidemiology"[All Fields] OR "prevalence"[All Fields] OR "prevalence"[MeSH Terms]))) OR ("hiv seroprevalence"[MeSH Terms] OR ("hiv"[All Fields] AND "seroprevalence"[All Fields]) OR "hiv seroprevalence"[All Fields])) OR ("hiv seropositivity"[MeSH Terms] OR ("hiv"[All Fields] AND "seropositivity"[All Fields]) OR "hiv seropositivity"[All Fields] OR ("hiv"[All Fields] AND "positivity"[All Fields]) OR "hiv positivity"[All Fields])	56,128
#5	Uptake[All Fields] OR (uptake[All Fields] AND care[All Fields])) OR (uptake[All Fields] AND testing[All Fields])) OR (uptake[All Fields] AND ("research design"[MeSH Terms] OR ("research"[All Fields] AND "design"[All Fields]) OR "research design"[All Fields] OR "test"[All Fields]))) OR (uptake[All Fields] AND ("hiv"[MeSH Terms] OR "hiv"[All Fields]) AND care[All Fields])) OR (uptake[All Fields] AND ("hiv"[MeSH Terms] OR "hiv"[All Fields]) AND testing[All Fields])) OR (uptake[All Fields] AND ("hiv"[MeSH Terms] OR "hiv"[All Fields]) AND ("research design"[MeSH Terms] OR ("research"[All Fields] AND "design"[All Fields]) OR "research design"[All Fields] OR "test"[All Fields]))) OR (("hiv"[MeSH Terms] OR "hiv"[All Fields]) AND testing[All Fields] AND uptake[All Fields])) OR (("hiv"[MeSH Terms] OR "hiv"[All Fields]) AND ("research design"[MeSH Terms] OR ("research"[All Fields] AND "design"[All Fields]) OR "research design"[All Fields] OR "test"[All Fields]) AND uptake[All Fields])) OR (("hiv"[MeSH Terms] OR "hiv"[All Fields]) AND care[All Fields] AND uptake[All Fields])	46,004
#4	(testing[All Fields] OR ("standard of care"[MeSH Terms] OR ("standard"[All Fields] AND "care"[All Fields]) OR "standard of care"[All Fields])) OR (provider-administered[All Fields] AND ("research design"[MeSH Terms] OR ("research"[All Fields] AND "design"[All Fields]) OR "research design"[All Fields] OR "test"[All Fields]))) OR (provider-initiated[All Fields] AND ("research design"[MeSH Terms] OR ("research"[All Fields] AND "design"[All Fields]) OR "research design"[All Fields] OR "test"[All Fields]))) OR (provider-initiated[All Fields] AND testing[All Fields])) OR (client-initiated[All Fields] AND testing[All Fields])) OR (client-initiated[All Fields] AND ("research design"[MeSH Terms] OR ("research"[All Fields] AND "design"[All Fields]) OR "research design"[All Fields] OR "test"[All Fields]))) OR (community-based[All Fields] AND ("hiv"[MeSH Terms] OR "hiv"[All Fields]) AND testing[All Fields])) OR (home-based[All Fields] AND ("hiv"[MeSH Terms] OR "hiv"[All Fields]) AND testing[All Fields])) OR (door-to-door-based[All Fields] AND ("hiv"[MeSH Terms] OR "hiv"[All Fields]) AND testing[All Fields])) OR (door-to-door-based[All Fields] AND ("hiv"[MeSH Terms] OR "hiv"[All Fields]) AND ("research design"[MeSH Terms] OR ("research"[All Fields] AND "design"[All Fields]) OR "research design"[All Fields] OR "test"[All Fields]))) OR (mobile[All Fields] AND ("hiv"[MeSH Terms] OR "hiv"[All Fields]) AND testing[All Fields])) OR (mobile[All Fields] AND ("hiv"[MeSH Terms] OR "hiv"[All Fields]) AND ("research design"[MeSH Terms] OR ("research"[All Fields] AND "design"[All Fields]) OR "research design"[All Fields] OR "test"[All Fields]))) OR (("hiv"[MeSH Terms] OR "hiv"[All Fields]) AND testing[All Fields] AND campaign[All Fields])) OR (bar-based[All Fields] AND ("hiv"[MeSH Terms] OR "hiv"[All Fields]) AND testing[All Fields])) OR (bar-based[All Fields] AND ("hiv"[MeSH Terms] OR "hiv"[All Fields]) AND ("research design"[MeSH Terms] OR ("research"[All Fields] AND "design"[All Fields]) OR "research design"[All Fields] OR "test"[All Fields]))) OR (("workplace"[MeSH Terms] OR "workplace"[All Fields]) AND ("hiv"[MeSH Terms] OR "hiv"[All Fields]) AND testing[All Fields])) OR (("workplace"[MeSH Terms] OR "workplace"[All Fields]) AND ("hiv"[MeSH Terms] OR "hiv"[All Fields]) AND ("research design"[MeSH Terms] OR ("research"[All Fields] AND "design"[All Fields]) OR "research design"[All Fields] OR "test"[All Fields]))) OR (church-based[All Fields] AND ("hiv"[MeSH Terms] OR "hiv"[All Fields]) AND testing[All Fields])) OR (church-based[All Fields] AND ("hiv"[MeSH Terms] OR "hiv"[All Fields]) AND ("research design"[MeSH Terms] OR ("research"[All Fields] AND "design"[All Fields]) OR "research design"[All Fields] OR "test"[All Fields]))	135,364
#3	Search #1 AND # 2	14,164
#2	“self[Text Word]) AND testing[All Fields])) OR (("hiv"[MeSH Terms] OR HIV[Text Word]) AND“(("ego"[MeSH Terms] OR self[Text Word]) AND testing[All Fields]))) OR ("diagnostic self evaluation"[MeSH Terms] OR diagnostic self-evaluation[Text Word])) OR ("self care"[MeSH Terms] OR self care[Text Word])) OR ("self administration"[MeSH Terms] OR self-administration[Text Word])) OR	14,302

	(("hiv"[MeSH Terms] OR HIV[Text Word]) AND ("ego"[MeSH Terms] OR self[Text Word]) AND ("diagnosis"[Subheading] OR "mass screening"[MeSH Terms] OR screening[Text Word]))	
#1	"adult"[MeSH Terms] OR (adult[All Fields] OR adulterant[All Fields] OR adulterate[All Fields] OR adulterated[All Fields] OR adulteration[All Fields] OR adulterations[All Fields] OR adultery[All Fields] OR adulthood [All Fields] OR adulticidal[All Fields] OR adultorum[All Fields] OR adults[All Fields])) OR ("young adult"[MeSH Terms] OR young adults[Text Word])) OR (young adult[All Fields] OR young adulthood [All Fields] OR young adults[All Fields])) OR ("middle aged "[MeSH Terms] OR middle aged [Text Word])) OR ("aged"[MeSH Terms] OR aged[Text Word])) OR ("aged, 80 and over"[MeSH Terms] OR aged, 80 and over[Text Word])	7,814,103

**Search in SCOPUS**		
**Search**	**Query**	**Items**
#14	Search # 3 AND # 11 AND # 12 AND # 13 AND (with publication date from Jan 1998 to Dec 2019)	406
#13	TITLE-ABS-KEY ("Africa" OR Africa? OR Algeria OR Angola OR Benin OR Botswana OR Burkina FasoOR Burundi OR Cameroon OR Canary Islands OR Cape Verde OR Central African Republic OR Chad OR Comoros OR Congo OR Democratic Republic of Congo OR Djibouti OR Egypt OR Equatorial Guinea OR Eritrea OR Ethiopia OR Gabon OR Gambia OR Ghana OR Guinea OR Guinea Bissau OR Ivory Coast OR Cote d'Ivoire OR Jamahiriya OR Jamahiryia OR Kenya OR Lesotho OR Liberia OR Libya OR Libia OR Madagascar OR Malawi OR Mali OR Mauritania OR Mauritius OR Mayote OR Morocco OR Mozambique OR Mocambique OR Namibia OR Niger OR Nigeria OR Principe OR Reunion OR Rwanda OR Sao Tome OR Senegal OR Seychelles OR Sierra Leone OR Somalia OR South Africa OR St Helena OR Sudan OR Swaziland OR Tanzania OR Togo OR Tunisia OR Uganda OR Western Sahara OR Zaire OR Zambia OR Zimbabwe OR Central Africa OR Central African OR West Africa OR West African OR Western Africa OR Western African OR East Africa OR East African OR Eastern Africa OR Eastern African OR North Africa OR North African OR Northern Africa OR Northern African OR South African OR Southern Africa OR Southern African OR sub Saharan Africa OR sub Saharan African OR subSaharan Africa OR subSaharan African NOT guinea pig OR guinea pigs OR aspergillus niger")	1,653,462
#12	TITLE-ABS-KEY ("randomized" AND "controlled" AND "trial " OR "controlled" AND "clinical trial" OR "randomized" OR "placebo" OR "drug" AND "therapy" OR "randomly" OR "trial" OR "groups" AND "quasi experimental" OR "after" OR "pre" AND "post" AND "research" AND "design" OR "test" OR "controlled" OR "interrupted time series analysis" OR "interrupted " AND "time" AND "series" OR "CBA").	44,624
#11	Search # 4 AND # 5 AND # 6 AND # 7 AND # 8 AND # 9 AND # 10	7
#10	TITLE-ABS-KEY ("self injurious behavior" OR "self-injurious" AND "behavior" OR "self-injurious" AND "behavior" OR "self-injurious" AND "behavior" OR "self" AND "injurious" AND "behavior" OR "self" AND "injurious" AND "behavior" OR "self" AND "harm" OR "self harm" OR "suicide" OR "suicide, attempted" OR "suicide" AND "attempted" OR "attempted suicide" OR "attempted" AND "suicide" OR "self " AND "destructive" AND "behavior" OR "self" AND "destructive" OR "suicidal ideation" OR "suicidal" AND "ideation" OR "suicidal" AND "behavior" OR "intimate partner violence" OR "intimate" AND "partner" AND "violence" OR "intimate" OR "partner" OR "violence" OR "spouse abuse" OR "spouse" AND "abuse" OR "spouse" OR "abuse")	29
#9	TITLE-ABS-KEY ("antigens" OR "antigen/cea" OR "antigen/factor" OR "antigen's" OR "antigenetic" OR "antigenic" OR "antigenically" OR "antigenicity" OR "antigenotoxic" OR "antigens/immunology" OR "antigensynthesis" OR " CD4" OR "t-lymphocytes" OR "T-Cell" OR "receptors" OR "cd4 antigens" OR "CD4 receptors")	7,045,282
#8	TITLE-ABS-KEY ("antiretroviral therapy, highly active" OR "anti-retroviral agents" OR "antiviral agents" OR "anti" AND "hiv" OR "antiretroviral" AND "retroviral" OR "HAART" OR "anti" AND "acquired AND immunodeficiency" OR "anti" AND "acquired AND immuno-deficiency" OR "anti" AND "acquired AND immune-deficiency" OR "anti" AND "acquired immue" AND "deficiency")	72,823
#7	TITLE-ABS-KEY ("hiv" OR "hiv" AND "linkage" OR "linkage" AND "hiv" OR "hiv" AND "care" OR "linkage" AND "hiv care" OR "link" AND " care" OR "link" AND "hiv" OR "link" AND "hiv care" OR "hiv" OR "hiv" AND "link")	11,691
#6	TITLE-ABS-KEY ("Yield" OR "hiv" OR "hiv" AND "epidemiology" OR "epidemiology" OR "prevalence" OR "hiv seroprevalence" OR "hiv" AND "seroprevalence" OR "hiv seroprevalence" OR "hiv seropositivity" OR "hiv" AND "seropositivity" OR "hiv seropositivity" OR "hiv" AND "positivity")	11,204

#5	TITLE-ABS-KEY (“Uptake” AND care” OR “uptake AND testing” OR “uptake AND research” AND “design" OR "research” AND “design" OR "research” AND “design” AND “test" OR “uptake” AND “hiv” OR "hiv” AND “care” OR “ uptake” AND “care”)	8,040
#4	TITLE-ABS-KEY (‘‘HIV’’ OR ‘‘Human AND Immunodeficiency AND virus’’ OR ‘‘AIDS AND Virus’’ OR ‘‘AIDS AND Viruses’’ OR ‘‘acquired AND immune AND deficiency AND syndrome’’ OR ‘‘Acquired AND Immunodeficiency AND Syndrome’’ OR ‘‘Human AND Immunodeficiency AND Virus’’ OR ‘‘Human AND Immunodeficiency AND Viruses’’ OR ‘‘Acquired AND Immune AND Deficiency AND Syndrome AND Virus’’)) AND (TITLE-ABS-KEY (‘‘self-test’’ OR ‘‘self-tests’’ OR ‘‘self-testing’’ OR ‘‘self-testers’’ OR ‘‘self-tested’’ OR ‘‘self-sampling’’ OR ‘‘self- AND examination’’ OR ‘‘diagnostic AND self AND evaluation’’ OR ‘‘self AND evaluation’’ OR ‘‘home AND test’’ OR ‘‘home tests’’ OR ‘‘home testing’’ OR ‘‘home-based test’’ OR ‘‘home- based AND tests’’ OR ‘‘home-based AND testing’’ OR ‘‘self-implemented’’)	1,910
#3	Search #1 AND # 2	37,237
#2	TITLE-ABS-KEY (‘‘self-test’’ OR ‘‘self-tests’’ OR ‘‘self-testing’’ OR ‘‘self- testers’’ OR ‘‘self-tested’’ OR ‘‘self-sampling’’ OR ‘‘self-examination’’ OR ‘‘diagnostic AND self AND evaluation’’ OR ‘‘self AND evaluation’’ OR ‘‘home AND test’’ OR ‘‘home AND tests’’ OR ‘‘home AND testing’’ OR ‘‘home-based AND test’’ OR ‘‘home-based AND tests’’ OR ‘‘home-based AND testing’’ OR ‘‘self-implemented’’)	57,044
#1	TITLE-ABS-KEY ("adult" OR "adulthood" OR "young AND adult" OR "young AND adulthood" OR "middle AND -aged")	11,174,853

Search in CDSR, CENTRAL, Cochrane Library		
**Search**	**Query**	**Items**
# 46	Search # 38 AND # 42 AND # 45 (with Cochrane Library publication date from Jan 1998 to Dec 2019)	543
# 45	# 43 OR # 44	89,864
# 44	(“Africa” OR “Africa* OR “Algeria” OR “Angola” OR “Benin” OR “Botswana” OR “Burkina Faso” OR “Burundi” OR “Cameroon” OR “Canary Islands” OR “Cape Verde” OR “Central African Republic” OR “Chad” OR “Comoros” OR “Congo” OR “Democratic Republic of Congo” OR “Djibouti” OR “Egypt” OR “Equatorial Guinea” OR “Eritrea” OR “Ethiopia” OR “Gabon” OR “Gambia” OR “Ghana” OR “Guinea” OR “Guinea Bissau” OR “Ivory Coast” OR “Cote d’Ivoire” OR “Jamahiriya” OR “Jamahiryia” OR “Kenya” OR “Lesotho” OR “Liberia” OR “Libya” OR “Libia” OR “Madagascar” OR “Malawi” OR “Mali” OR “Mauritania” OR “Mauritius” OR “Mayotte” OR “Morocco” OR “Mozambique” OR “Mocambique” OR “Namibia” OR “Niger” OR “Nigeria” OR “Principe” OR “Reunion” OR “Rwanda” OR “Sao Tome” OR “Senegal” OR “Seychelles” OR “Sierra Leone” OR “Somalia” OR “South Africa” OR “St Helena” OR “Sudan” OR “Swaziland” OR “Tanzania” OR “Togo” OR “Tunisia” OR “Uganda” OR “Western Sahara” OR “Zaire” OR “Zambia” OR “Zimbabwe” OR “Central Africa” OR “Central African “ OR “West Africa” OR “West African” OR “Western Africa” OR “Western African” OR “East Africa” OR “East African” OR “Eastern Africa” OR “Eastern African” OR “North Africa” OR “North African” OR “Northern Africa” OR “Northern African” OR “South African” OR “Southern Africa” OR “Southern African” OR “sub Saharan Africa” OR “sub Saharan African” OR “subSaharan Africa” OR “subSaharan African” NOT “guinea pig” OR “guinea pigs” OR “aspergillus niger”)	9,962
# 43	MesH Descriptor: [Africa] explode all trees	6,700
# 42	# 39 OR # 40 OR # 41	161,648
# 41	("randomized" AND "controlled" AND "trial " OR "controlled" AND "clinical trial" OR "randomized" OR "placebo" OR "drug" AND "therapy" OR "randomly" OR "trial" OR "groups" AND "quasi experimental" OR "after" OR "pre" AND "post" AND "research" AND "design" OR "test" OR "controlled" OR "interrupted time series analysis" OR "interrupted " AND "time" AND "series" OR "CBA").	1,632,181
# 40	MesH Descriptor: [Interrupted Time Series Analysis] explode all trees	33

# 39	MesH Descriptor: [Quasi Experimental] explode all trees	60
# 38	Search # 4 AND #10 AND # 12 AND # 17 AND # 20 AND # 24 AND # 28 AND # 37	726
# 37	# 29 OR # 30 OR # 31 OR # 32 OR # 33 OR # 34 OR # 35 OR # 36	1,375,121
# 36	("self injurious behavior" OR "self-injurious" AND "behavior" OR "self-injurious" AND "behavior" OR "self-injurious" AND "behavior" OR "self" AND "injurious" AND "behavior" OR "self" AND "injurious" AND "behavior" OR "self" AND "harm" OR "self harm" OR "suicide" OR "suicide, attempted" OR "suicide" AND "attempted" OR "attempted suicide" OR "attempted" AND "suicide" OR "self " AND "destructive" AND "behavior" OR "self" AND "destructive" OR "suicidal ideation" OR "suicidal" AND "ideation" OR "suicidal" AND "behavior" OR "intimate partner violence" OR "intimate" AND "partner" AND "violence" OR "intimate" OR "partner" OR "violence" OR "spouse abuse" OR "spouse" AND "abuse" OR "spouse" OR "abuse") : ti,ab,kw (Word variations have been searched)	85,318
# 35	MesH Descriptor: [Spouse Abuse] explode all trees	194
# 34	MesH Descriptor: [Intimate Partner Violence] explode all trees	300
# 33	MesH Descriptor: [Behavior] explode all trees	2
# 32	MesH Descriptor: [Suicidal Ideation] explode all trees	437
# 31	MesH Descriptor: [Suicide, Attempted] explode all trees	401
# 30	MesH Descriptor: [Suicide] explode all trees	1,158
# 29	MesH Descriptor: [Self-Injurious Behavior] explode all trees	1,363
# 28	#25 OR #26 OR #27	300,543
#27	("antigens" OR "antigen/cea" OR "antigen/factor" OR "antigen's" OR "antigenetic" OR "antigenic" OR "antigenically" OR "antigenicity" OR "antigenotoxic" OR "antigens/immunology" OR "antigensynthesis" OR " CD4" OR "t-lymphocytes" OR "T-Cell" OR "receptors" OR "cd4 antigens" OR "CD4 receptors") : ti,ab,kw (Word variations have been searched)	76,455
#26	MesH Descriptor: [T-Lymphocytes] explode all trees	3,276
#25	MesH Descriptor: [Antigens] explode all trees	14,056
#24	#21 OR #22 OR #23	424,061
#23	("antiretroviral therapy, highly active" OR "anti-retroviral agents" OR "antiviral agents" OR "anti" AND "hiv" OR "antiretroviral" AND "retroviral" OR "HAART" OR "anti" AND "acquired AND immunodeficiency" OR "anti" AND "acquired AND immuno-deficiency" OR "anti" AND "acquired AND immune-deficiency" OR "anti" AND "acquired immue" AND "deficiency") : ti,ab,kw (Word variations have been searched)	11,130
#22	MesH Descriptor: [anti-retroviral agents] explode all trees	4,370
#21	MesH Descriptor: [Antiretroviral Therapy, Highly Active] explode all trees	1,179
#20	# 18 OR # 19	414,502
#19	("hiv" OR "hiv" AND "linkage" OR "linkage" AND "hiv" OR "hiv" AND "care" OR "linkage" AND "hiv care" OR "link" AND " care" OR "link" AND "hiv" OR "link" AND "hiv care" OR "hiv" OR "hiv" AND "link") : ti,ab,kw (Word variations have been searched)	263,625
#18	MesH Descriptor: [Genetic Linkage] explode all trees	178
#17	# 13 OR # 14 OR #15 OR # 16	522,615
#16	("Yield" OR "hiv" OR "hiv" AND "epidemiology" OR "epidemiology" OR "prevalence" OR "hiv seroprevalence" OR "hiv" AND "seroprevalence" OR "hiv seroprevalence" OR "hiv seropositivity" OR "hiv" AND "seropositivity" OR "hiv seropositivity" OR "hiv" AND "positivity") : ti,ab,kw (Word variations have been searched)	115,925
#15	MesH Descriptor: [hiv seropositivity] explode all trees	724
#14	MesH Descriptor: [Seroprevalence] explode all trees	114
#13	MesH Descriptor: [Prevalence] explode all trees	4,459
#12	("Uptake" AND care" OR "uptake AND testing" OR "uptake AND research" AND "design" OR "research" AND "design" OR "research" AND "design" AND "test" OR "uptake" AND "hiv" OR "hiv" AND "care" OR " uptake" AND "care") : ti,ab,kw (Word variations have been searched)	829,908
#11	MesH Descriptor: [Research Design] explode all trees	159,219
#10	(''HIV'' OR ''Human AND Immunodeficiency AND virus'' OR ''AIDS AND Virus'' OR ''AIDS AND Viruses'' OR ''acquired AND immune AND deficiency AND syndrome'' OR ''Acquired AND Immunodeficiency AND Syndrome'' OR ''Human AND Immunodeficiency AND Virus'' OR ''Human	28,030

	AND Immunodeficiency AND Viruses'' OR ''Acquired AND Immune AND Deficiency AND Syndrome AND Virus'') : ti,ab,kw (Word variations have been searched)	
#9	MesH Descriptor: [Acquired Immunodeficiency Syndrome] explode all trees	1923
# 8	MesH Descriptor: [HIV] explode all trees	2984
#7	(''self-test'' OR ''self-tests'' OR ''self-testing'' OR ''self- testers'' OR ''self-tested'' OR ''self-sampling'' OR ''self-examination'' OR ''diagnostic self evaluation'' OR ''self evaluation'' OR ''home test'' OR ''home tests'' OR ''home testing'' OR ''home-based test'' OR ''home-based tests'' OR ''home-based testing'' OR ''self-implemented'') ti,ab,kw (Word variations have been searched)	3,202
#6	MesH Descriptor: [Self-examination] explode all trees	199
#5	MesH Descriptor: [Diagnostic Self Evaluation] explode all trees	174
#4	#1 OR #2 OR #3	27,769
#3	("adult" OR "adulthood" OR "young adult" OR "young adulthood" OR "middle -aged"): ti,ab,kw(Word variations have been searched)	660,968
#2	MesH Descriptor: [Young Adult] explode all trees	4,108
#1	MesH Descriptor: [Adult] explode all trees	18,623
**Search in MEDLINE,EBSCOhost**		
**Search**	**Query**	**Items**
# 46	Search # 38 AND # 42 AND # 45 (with publication date from Jan 1998 to Dec 2019)	47
# 45	or /43 -44	217,322
# 44	(Africa OR Africa? OR Algeria OR Angola OR Benin OR Botswana OR Burkina FasoOR Burundi OR Cameroon OR Canary Islands OR Cape Verde OR Central African Republic OR Chad OR Comoros OR Congo OR Democratic Republic of Congo OR Djibouti OR Egypt OR Equatorial Guinea OR Eritrea OR Ethiopia OR Gabon OR Gambia OR Ghana OR Guinea OR Guinea Bissau OR Ivory Coast OR Cote d'Ivoire OR Jamahiriya OR Jamahiryia OR Kenya OR Lesotho OR Liberia OR Libya OR Libia OR Madagascar OR Malawi OR Mali OR Mauritania OR Mauritius OR Mayote OR Morocco OR Mozambique OR Mocambique OR Namibia OR Niger OR Nigeria OR Principe OR Reunion OR Rwanda OR Sao Tome OR Senegal OR Seychelles OR Sierra Leone OR Somalia OR South Africa OR St Helena OR Sudan OR Swaziland OR Tanzania OR Togo OR Tunisia OR Uganda OR Western Sahara OR Zaire OR Zambia OR Zimbabwe OR Central Africa OR Central African OR West Africa OR West African OR Western Africa OR Western African OR East Africa OR East African OR Eastern Africa OR Eastern African OR North Africa OR North African OR Northern Africa OR Northern African OR South African OR Southern Africa OR Southern African OR sub Saharan Africa OR sub Saharan African OR subSaharan Africa OR subSaharan African NOT guinea pig OR guinea pigs OR aspergillus niger)	21
# 43	Africa/	217,318
# 42	or/39 -41	9,612,199
# 41	(randomized AND controlled AND trial OR controlled AND clinical trial OR randomized OR placebo OR drug AND therapy OR randomly OR trial OR groups AND quasi experimental OR after OR pre AND post AND research AND design OR test OR controlled OR interrupted time series analysis OR interrupted AND time AND series OR CBA).	9,610,250
# 40	Interrupted Time Series Analysis/	2,065
# 39	Quasi Experimental/	13,365
# 38	Search # 4 AND #10 AND # 12 AND # 17 AND # 20 AND # 24 AND # 28 AND # 37	404
# 37	or /29 -36	1,706,833

# 36	(self injurious behavior OR self-injurious AND behavior OR self-injurious AND behavior OR self-injurious AND behavior OR self AND injurious AND behavior OR self AND injurious AND behavior OR self AND harm OR self harm OR suicide OR suicide, attempted OR "suicide" AND "attempted" OR "attempted suicide" OR "attempted" AND "suicide" OR "self " AND destructive AND behavior OR self AND destructive OR suicidal ideation OR suicidal AND ideation OR suicidal AND behavior OR intimate partner violence OR intimate AND partner AND violence OR intimate OR partner OR violence OR spouse abuse OR spouse AND abuse OR spouse OR abuse) : ti,ab,kf	508,956
# 35	Spouse Abuse/	9,343
# 34	Intimate Partner Violence/	12,677
# 33	Behavior/	1,304,479
# 32	Suicidal Ideation/	13,387
# 31	Suicide, Attempted/	21,843
# 30	Suicide/	87,413
# 29	Self-Injurious Behavior/	8,623
# 28	or/25 -27	2,728,140
#27	(antigens OR antigen/cea OR antigen/factor OR antigen's OR antigenetic OR antigenic OR antigenically OR antigenicity OR antigenotoxic OR antigens/immunology OR antigensynthesis OR CD4 OR t-lymphocytes OR T-Cell OR receptors OR cd4 antigens OR CD4 receptors) : ti,ab,kf	2,728,137
#26	T-Lymphocytes/	331,996
#25	Antigens/	986,296
#24	or /21 -23	172,730
#23	(antiretroviral therapy, highly active OR anti-retroviral agents OR antiviral agents OR anti AND hiv OR antiretroviral AND retroviral OR HAART OR anti AND acquired AND immunodeficiency OR anti AND acquired AND immuno-deficiency OR anti AND acquired AND immune-deficiency OR anti AND acquired immue AND deficiency) : ti,ab,kf	172,723
#22	Anti-retroviral agents/	10,479
#21	Antiretroviral Therapy, Highly Active/	25,578
#20	or /18 -19	426,041
#19	(hiv OR hiv AND linkage OR linkage AND hiv OR hiv AND care OR linkage AND hiv care OR link AND care OR link AND hiv OR link AND hiv care OR hiv OR hiv AND link) : ti,ab,kf	380,284
#18	Genetic Linkage/	45,856
#17	or /13 -16	2,897,528
#16	(Yield OR hiv OR hiv AND epidemiology OR epidemiology OR prevalence OR hiv seroprevalence OR hiv AND seroprevalence OR hiv seroprevalence OR hiv seropositivity OR hiv AND seropositivity OR hiv seropositivity OR hiv AND positivity) : ti,ab,kf	2,895,218
#15	hiv seropositivity/	23,982
#14	Seroprevalence/	20,632
#13	Prevalence/	708,082
#12	(Uptake AND care OR uptake AND testing OR uptake AND research AND design OR research AND design OR research AND design AND test OR uptake AND hiv OR hiv AND care OR uptake AND care) : ti,ab,kf	447,335
#11	Research Design/	133,906
#10	(HIV OR Human AND Immunodeficiency AND virus OR AIDS AND Virus OR AIDS AND Viruses OR acquired AND immune AND deficiency AND syndrome OR Acquired AND Immunodeficiency AND Syndrome OR Human AND Immunodeficiency AND Virus OR Human AND Immunodeficiency AND Viruses OR Acquired AND Immune AND Deficiency AND Syndrome AND Virus) : ti,ab,kf	426,127
#9	Acquired Immunodeficiency Syndrome/	89,948
# 8	HIV/	360,797
#7	(self-test OR self-tests OR self-testing OR self- testers OR self-tested OR self-sampling OR self-examination OR diagnostic self evaluation OR self evaluation OR home test OR home tests OR home testing OR home-based test OR home-based tests OR home-based testing OR self-implemented) ti,ab,kf	4,360
#6	Self-examination/	4,349

#5	Diagnostic Self Evaluation/	3,285
#4	or /1-3	7,198,340
#3	(adult OR adulthood OR young adult OR young adulthood OR middle -aged). ti,ab,kf	7,198,340
#2	Young Adult/	905,960
#1	Adult/	5,813,687

**Search in CINAHL, EBSCOhost**		
**Search**	**Query**	**Items**
S 48	Search S 40 AND S 44 AND S 47 (with publication date from Jan 1998 to Dec 2019)	15
S 47	S 45 OR S 46	104,605
S 46	(Africa OR Algeria OR Angola OR Benin OR Botswana OR Burkina FasoOR Burundi OR Cameroon OR Canary Islands OR Cape Verde OR Central African Republic OR Chad OR Comoros OR Congo OR Democratic Republic of Congo OR Djibouti OR Egypt OR Equatorial Guinea OR Eritrea OR Ethiopia OR Gabon OR Gambia OR Ghana OR Guinea OR Guinea Bissau OR Ivory Coast OR Cote d'Ivoire OR Jamahiriya OR Jamahiryia OR Kenya OR Lesotho OR Liberia OR Libya OR Libia OR Madagascar OR Malawi OR Mali OR Mauritania OR Mauritius OR Mayote OR Morocco OR Mozambique OR Mocambique OR Namibia OR Niger OR Nigeria OR Principe OR Reunion OR Rwanda OR Sao Tome OR Senegal OR Seychelles OR Sierra Leone OR Somalia OR South Africa OR St Helena OR Sudan OR Swaziland OR Tanzania OR Togo OR Tunisia OR Uganda OR Western Sahara OR Zaire OR Zambia OR Zimbabwe OR Central Africa OR Central African OR West Africa OR West African OR Western Africa OR Western African OR East Africa OR East African OR Eastern Africa OR Eastern African OR North Africa OR North African OR Northern Africa OR Northern African OR South African OR Southern Africa OR Southern African OR sub Saharan Africa OR sub Saharan African OR subSaharan Africa OR subSaharan African NOT guinea pig OR guinea pigs OR aspergillus niger)	104,568
S 45	(MH "Africa")	44,956
S 44	S 41 OR S 42 OR S 43	2,163,474
S 43	(randomized AND controlled AND trial OR controlled AND clinical trial OR randomized OR placebo OR drug AND therapy OR randomly OR trial OR groups AND quasi experimental OR after OR pre AND post AND research AND design OR test OR controlled OR interrupted time series analysis OR interrupted AND time AND series OR CBA).	2,161,336
S 42	(MH "Interrupted Time Series Analysis")	808
S 41	(MH "Quasi Experimental")	16,770
S 40	Search S 4 AND S8 AND S14 AND S19 AND S22 AND S26 AND S30 AND S39	3
S 39	S 31 OR S 32 OR S 33 OR S 34 OR S 35 OR S 36 OR S 37 OR S 38	515,405
S 38	(self injurious behavior OR self-injurious AND behavior OR self-injurious AND behavior OR self-injurious AND behavior OR self AND injurious AND behavior OR self AND injurious AND behavior OR self AND harm OR self harm OR suicide OR suicide, attempted OR "suicide" AND "attempted" OR "attempted suicide" OR "attempted" AND "suicide" OR "self " AND destructive AND behavior OR self AND destructive OR suicidal ideation OR suicidal AND ideation OR suicidal AND behavior OR intimate partner violence OR intimate AND partner AND violence OR intimate OR partner OR violence OR spouse abuse OR spouse AND abuse OR spouse OR abuse)	250,490
S 37	(MH "Spouse Abuse")	8,658
S 36	(MH "Intimate Partner Violence")	16,972
S 35	(MH "Behavior")	311,355
S 34	(MH "Suicidal Ideation")	9,512
S 33	(MH "Suicide, Attempted")	6,896
S 32	(MH "Suicide")	33,403
S 31	(MH "Self-Injurious Behavior")	4,509

S 30	S 27 OR S 28 OR S 29	184,800
S 29	(antigens OR antigen/cea OR antigen/factor OR antigen's OR antigenetic OR antigenic OR antigenically OR antigenicity OR antigenotoxic OR antigens/immunology OR antigensynthesis OR CD4 OR t-lymphocytes OR T-Cell OR receptors OR cd4 antigens OR CD4 receptors)	184,714
S 28	(MH “T-Lymphocytes”)	16,128
S 27	(MH “Antigens”)	31,761
S 26	S 23 OR S 24 OR S 25	40,000
S 25	(antiretroviral therapy, highly active OR anti-retroviral agents OR antiviral agents OR anti AND hiv OR antiretroviral AND retroviral OR HAART OR anti AND acquired AND immunodeficiency OR anti AND acquired AND immuno-deficiency OR anti AND acquired AND immune-deficiency OR anti AND acquired immue AND deficiency)	39,983
S 24	(MH “Anti-retroviral agents”)	5,769
S 23	(MH “Antiretroviral Therapy, Highly Active”)	6,880
S 22	S 20 OR S 21	120,646
S 21	(hiv OR hiv AND linkage OR linkage AND hiv OR hiv AND care OR linkage AND hiv care OR link AND care OR link AND hiv OR link AND hiv care OR hiv OR hiv AND link)	120,421
S 20	(MH “Genetic Linkage”)	230
S 19	S 15 OR S 16 OR S 17 S 18	634,784
S 18	(Yield OR hiv OR hiv AND epidemiology OR epidemiology OR prevalence OR hiv seroprevalence OR hiv AND seroprevalence OR hiv seroprevalence OR hiv seropositivity OR hiv AND seropositivity OR hiv seropositivity OR hiv AND positivity)	634,311
S 17	(MH “hiv seropositivity”)	4,787
S 16	(MH “Seroprevalence”)	2,828
S 15	(MH “Prevalence”)	214,403
S 14	S 9 OR S 10 OR S 11 OR S 12 OR S 13	325,209
S 13	(Uptake AND care OR uptake AND testing OR uptake AND research AND design OR research AND design OR research AND design AND test OR uptake AND hiv OR hiv AND care OR uptake AND care)	236,755
S 12	(MH “Research Design”)	33,369
S 11	(HIV OR Human AND Immunodeficiency AND virus OR AIDS AND Virus OR AIDS AND Viruses OR acquired AND immune AND deficiency AND syndrome OR Acquired AND Immunodeficiency AND Syndrome OR Human AND Immunodeficiency AND Virus OR Human AND Immunodeficiency AND Viruses OR Acquired AND Immune AND Deficiency AND Syndrome AND Virus)	114,336
S 10	(MH “Acquired Immunodeficiency Syndrome”)	12,466
S 9	(MH “HIV”)	109,904
S 8	S 5 OR S 6 OR S 7	24,801
S 7	(self-test OR self-tests OR self-testing OR self- testers OR self-tested OR self-sampling OR self-examination OR diagnostic self evaluation OR self evaluation OR home test OR home tests OR home testing OR home-based test OR home-based tests OR home-based testing OR self-implemented)	23,607
S 6	(MH “Self-examination”)	3,084
S 5	(MH “Diagnostic Self Evaluation”)	52,171
S 4	S1 OR S 2	6,793,800
S 3	(“adult OR adulthood OR young adult OR young adulthood OR middle –aged”).	34,478
S 2	(MH “Young Adult”)	273,092
S1	(MH “Adult”)	6,793,795

Search in Africa-Wide-Information, EBSCOhost		
**Search**	**Query**	**Items**
S 54	S8 AND S13 AND S18 AND S23 AND S28 AND S33 AND S38 AND S43 AND S48 AND S53	2
S 53	S 49 OR S 50 OR S 51 OR S 52	443,879
S 52	TI((randomized AND controlled AND trial OR controlled AND clinical trial OR randomized OR placebo OR drug AND therapy OR randomly OR trial OR groups AND quasi experimental OR after OR pre AND post AND research AND design OR test OR controlled OR interrupted time series analysis OR interrupted AND time AND series OR CBA) OR AB ((randomized AND controlled AND trial OR controlled AND clinical trial OR randomized OR placebo OR drug AND therapy OR randomly OR trial OR groups AND quasi experimental OR after OR pre AND post AND research AND design OR test OR controlled OR interrupted time series analysis OR interrupted AND time AND series OR CBA)) OR AB((randomized AND controlled AND trial OR controlled AND clinical trial OR randomized OR placebo OR drug AND therapy OR randomly OR trial OR groups AND quasi experimental OR after OR pre AND post AND research AND design OR test OR controlled OR interrupted time series analysis OR interrupted AND time AND series OR CBA))	384,405
S 51	SU(randomized AND controlled AND trial OR controlled AND clinical trial OR randomized OR placebo OR drug AND therapy OR randomly OR trial OR groups AND quasi experimental OR after OR pre AND post AND research AND design OR test OR controlled OR interrupted time series analysis OR interrupted AND time AND series OR CBA)	69,877
S50	SM(randomized AND controlled AND trial OR controlled AND clinical trial OR randomized OR placebo OR drug AND therapy OR randomly OR trial OR groups AND quasi experimental OR after OR pre AND post AND research AND design OR test OR controlled OR interrupted time series analysis OR interrupted AND time AND series OR CBA)	9
S 49	KW(randomized AND controlled AND trial OR controlled AND clinical trial OR randomized OR placebo OR drug AND therapy OR randomly OR trial OR groups AND quasi experimental OR after OR pre AND post AND research AND design OR test OR controlled OR interrupted time series analysis OR interrupted AND time AND series OR CBA)	101,549
S 48	S 44 OR S 45 OR S 46 OR S 47	65,819
S 47	TI ((self injurious behavior OR self-injurious AND behavior OR self-injurious AND behavior OR self-injurious AND behavior OR self AND injurious AND behavior OR self AND injurious AND behavior OR self AND harm OR self harm OR suicide OR suicide, attempted OR "suicide" AND "attempted" OR "attempted suicide" OR "attempted" AND "suicide" OR "self " AND destructive AND behavior OR self AND destructive OR suicidal ideation OR suicidal AND ideation OR suicidal AND behavior OR intimate partner violence OR intimate AND partner AND violence OR intimate OR partner OR violence OR spouse abuse OR spouse AND abuse OR spouse OR abuse)) OR AB ((self injurious behavior OR self-injurious AND behavior OR self-injurious AND behavior OR self-injurious AND behavior OR self AND injurious AND behavior OR self AND injurious AND behavior OR self AND harm OR self harm OR suicide OR suicide, attempted OR "suicide" AND "attempted" OR "attempted suicide" OR "attempted" AND "suicide" OR "self " AND destructive AND behavior OR self AND destructive OR suicidal ideation OR suicidal AND ideation OR suicidal AND behavior OR intimate partner violence OR intimate AND partner AND violence OR intimate OR partner OR violence OR spouse abuse OR spouse AND abuse OR spouse OR abuse)) OR AB ((self injurious behavior OR self-injurious AND behavior OR self-injurious AND behavior OR self-injurious AND behavior OR self AND injurious AND behavior OR self AND injurious AND behavior OR self AND harm OR self harm OR suicide OR suicide, attempted OR "suicide" AND "attempted" OR "attempted suicide" OR "attempted" AND "suicide" OR "self " AND destructive AND behavior OR self AND destructive OR suicidal ideation OR suicidal AND ideation OR suicidal AND behavior OR intimate partner violence OR intimate AND partner AND violence OR intimate OR partner OR violence OR spouse abuse OR spouse AND abuse OR spouse OR abuse))	49,858
S 46	SU(self injurious behavior OR self-injurious AND behavior OR self-injurious AND behavior OR self-injurious AND behavior OR self AND injurious AND behavior OR self AND injurious AND behavior OR self AND harm OR self harm OR suicide OR suicide, attempted OR "suicide" AND "attempted" OR "attempted suicide" OR "attempted" AND "suicide" OR "self " AND destructive AND behavior OR self AND destructive OR suicidal ideation OR suicidal AND ideation OR suicidal AND behavior OR intimate partner violence OR intimate AND partner AND violence OR intimate OR partner OR violence OR	25,495

	spouse abuse OR spouse AND abuse OR spouse OR abuse)	
S 45	SM(self injurious behavior OR self-injurious AND behavior OR self-injurious AND behavior OR self-injurious AND behavior OR self AND injurious AND behavior OR self AND injurious AND behavior OR self AND harm OR self harm OR suicide OR suicide, attempted OR "suicide" AND "attempted" OR "attempted suicide" OR "attempted" AND "suicide" OR "self " AND destructive AND behavior OR self AND destructive OR suicidal ideation OR suicidal AND ideation OR suicidal AND behavior OR intimate partner violence OR intimate AND partner AND violence OR intimate OR partner OR violence OR spouse abuse OR spouse AND abuse OR spouse OR abuse)	274
S 44	KW(self injurious behavior OR self-injurious AND behavior OR self-injurious AND behavior OR self-injurious AND behavior OR self AND injurious AND behavior OR self AND injurious AND behavior OR self AND harm OR self harm OR suicide OR suicide, attempted OR "suicide" AND "attempted" OR "attempted suicide" OR "attempted" AND "suicide" OR "self " AND destructive AND behavior OR self AND destructive OR suicidal ideation OR suicidal AND ideation OR suicidal AND behavior OR intimate partner violence OR intimate AND partner AND violence OR intimate OR partner OR violence OR spouse abuse OR spouse AND abuse OR spouse OR abuse)	29,341
S 43	S39 OR S40 OR S41 OR S42	80,794
S 42	TI((antigens OR antigen/cea OR antigen/factor OR antigen's OR antigenetic OR antigenic OR antigenically OR antigenicity OR antigenotoxic OR antigens/immunology OR antigensynthesis OR CD4 OR t-lymphocytes OR T-Cell OR receptors OR cd4 antigens OR CD4 receptors)) OR AB ((antigens OR antigen/cea OR antigen/factor OR antigen's OR antigenetic OR antigenic OR antigenically OR antigenicity OR antigenotoxic OR antigens/immunology OR antigensynthesis OR CD4 OR t-lymphocytes OR T-Cell OR receptors OR cd4 antigens OR CD4 receptors)) OR AB ((antigens OR antigen/cea OR antigen/factor OR antigen's OR antigenetic OR antigenic OR antigenically OR antigenicity OR antigenotoxic OR antigens/immunology OR antigensynthesis OR CD4 OR t-lymphocytes OR T-Cell OR receptors OR cd4 antigens OR CD4 receptors))	65,628
S41	SU(antigens OR antigen/cea OR antigen/factor OR antigen's OR antigenetic OR antigenic OR antigenically OR antigenicity OR antigenotoxic OR antigens/immunology OR antigensynthesis OR CD4 OR t-lymphocytes OR T-Cell OR receptors OR cd4 antigens OR CD4 receptors)	36,009
S40	SM (antigens OR antigen/cea OR antigen/factor OR antigen's OR antigenetic OR antigenic OR antigenically OR antigenicity OR antigenotoxic OR antigens/immunology OR antigensynthesis OR CD4 OR t-lymphocytes OR T-Cell OR receptors OR cd4 antigens OR CD4 receptors)	36,194
S39	KW(antigens OR antigen/cea OR antigen/factor OR antigen's OR antigenetic OR antigenic OR antigenically OR antigenicity OR antigenotoxic OR antigens/immunology OR antigensynthesis OR CD4 OR t-lymphocytes OR T-Cell OR receptors OR cd4 antigens OR CD4 receptors)	36,645
S38	S 34 OR S35 OR 36 OR 37	16,310
S37	TI((antiretroviral therapy, highly active OR anti-retroviral agents OR antiviral agents OR anti AND hiv OR antiretroviral AND retroviral OR HAART OR anti AND acquired AND immunodeficiency OR anti AND acquired AND immuno-deficiency OR anti AND acquired AND immune-deficiency OR anti AND acquired immue AND deficiency)) OR AB ((antiretroviral therapy, highly active OR anti-retroviral agents OR antiviral agents OR anti AND hiv OR antiretroviral AND retroviral OR HAART OR anti AND acquired AND immunodeficiency OR anti AND acquired AND immuno-deficiency OR anti AND acquired AND immune-deficiency OR anti AND acquired immue AND deficiency)) OR AB ((antiretroviral therapy, highly active OR anti-retroviral agents OR antiviral agents OR anti AND hiv OR antiretroviral AND retroviral OR HAART OR anti AND acquired AND immunodeficiency OR anti AND acquired AND immuno-deficiency OR anti AND acquired AND immune-deficiency OR anti AND acquired immue AND deficiency))	8,254
S36	SU(antiretroviral therapy, highly active OR anti-retroviral agents OR antiviral agents OR anti AND hiv OR antiretroviral AND retroviral OR HAART OR anti AND acquired AND immunodeficiency OR anti AND acquired AND immuno-deficiency OR anti AND acquired AND immune-deficiency OR anti AND acquired immue AND deficiency)	10,005
S35	SM(antiretroviral therapy, highly active OR anti-retroviral agents OR antiviral agents OR anti AND hiv OR antiretroviral AND retroviral OR HAART OR anti AND acquired AND immunodeficiency OR anti AND acquired AND immuno-deficiency OR anti AND acquired AND immune-deficiency OR anti AND acquired immue AND deficiency)	1
S34	KW(antiretroviral therapy, highly active OR anti-retroviral agents OR antiviral agents OR anti AND hiv OR antiretroviral AND retroviral OR HAART OR anti AND acquired AND immunodeficiency OR anti AND acquired AND immuno-deficiency OR anti AND acquired AND immune-deficiency OR anti AND acquired immue AND deficiency)	10,012
S33	S29 OR S30 OR S31 OR S32	109,045

S32	TI((hiv OR hiv AND linkage OR linkage AND hiv OR hiv AND care OR linkage AND hiv care OR link AND care OR link AND hiv OR link AND hiv care OR hiv OR hiv AND link)) OR AB ((hiv OR hiv AND linkage OR linkage AND hiv OR hiv AND care OR linkage AND hiv care OR link AND care OR link AND hiv OR link AND hiv care OR hiv OR hiv AND link)) OR AB ((hiv OR hiv AND linkage OR linkage AND hiv OR hiv AND care OR linkage AND hiv care OR link AND care OR link AND hiv OR link AND hiv care OR hiv OR hiv AND link))	94,426
S31	SU (hiv OR hiv AND linkage OR linkage AND hiv OR hiv AND care OR linkage AND hiv care OR link AND care OR link AND hiv OR link AND hiv care OR hiv OR hiv AND link)	59,107
S30	SM (hiv OR hiv AND linkage OR linkage AND hiv OR hiv AND care OR linkage AND hiv care OR link AND care OR link AND hiv OR link AND hiv care OR hiv OR hiv AND link)	5,449
S29	KW (hiv OR hiv AND linkage OR linkage AND hiv OR hiv AND care OR linkage AND hiv care OR link AND care OR link AND hiv OR link AND hiv care OR hiv OR hiv AND link)	57,952
S28	S24 OR S25 OR S26 OR S27	275,729
S27	TI ((Yield OR hiv OR hiv AND epidemiology OR epidemiology OR prevalence OR hiv seroprevalence OR hiv AND seroprevalence OR hiv seroprevalence OR hiv seropositivity OR hiv AND seropositivity OR hiv seropositivity OR hiv AND positivity)) OR AB ((Yield OR hiv OR hiv AND epidemiology OR epidemiology OR prevalence OR hiv seroprevalence OR hiv AND seroprevalence OR hiv seroprevalence OR hiv seropositivity OR hiv AND seropositivity OR hiv seropositivity OR hiv AND positivity)) OR AB ((Yield OR hiv OR hiv AND epidemiology OR epidemiology OR prevalence OR hiv seroprevalence OR hiv AND seroprevalence OR hiv seroprevalence OR hiv seropositivity OR hiv AND seropositivity OR hiv seropositivity OR hiv AND positivity))	204,661
S26	SU(Yield OR hiv OR hiv AND epidemiology OR epidemiology OR prevalence OR hiv seroprevalence OR hiv AND seroprevalence OR hiv seroprevalence OR hiv seropositivity OR hiv AND seropositivity OR hiv seropositivity OR hiv AND positivity)	140,971
S25	SM(Yield OR hiv OR hiv AND epidemiology OR epidemiology OR prevalence OR hiv seroprevalence OR hiv AND seroprevalence OR hiv seroprevalence OR hiv seropositivity OR hiv AND seropositivity OR hiv seropositivity OR hiv AND positivity)	7,041
S24	KW(Yield OR hiv OR hiv AND epidemiology OR epidemiology OR prevalence OR hiv seroprevalence OR hiv AND seroprevalence OR hiv seroprevalence OR hiv seropositivity OR hiv AND seropositivity OR hiv seropositivity OR hiv AND positivity)	145,846
S23	S 19 OR S 20 OR S 21 OR S 22	38,373
S 22	TI((Uptake AND care OR uptake AND testing OR uptake AND research AND design OR research AND design OR research AND design AND test OR uptake AND hiv OR hiv AND care OR uptake AND care)) OR AB ((Uptake AND care OR uptake AND testing OR uptake AND research AND design OR research AND design OR research AND design AND test OR uptake AND hiv OR hiv AND care OR uptake AND care)) OR AB ((Uptake AND care OR uptake AND testing OR uptake AND research AND design OR research AND design OR research AND design AND test OR uptake AND hiv OR hiv AND care OR uptake AND care))	31,783
S 21	SU (Uptake AND care OR uptake AND testing OR uptake AND research AND design OR research AND design OR research AND design AND test OR uptake AND hiv OR hiv AND care OR uptake AND care)	10,872
S 20	SM (Uptake AND care OR uptake AND testing OR uptake AND research AND design OR research AND design OR research AND design AND test OR uptake AND hiv OR hiv AND care OR uptake AND care)	27
S 19	KW (Uptake AND care OR uptake AND testing OR uptake AND research AND design OR research AND design OR research AND design AND test OR uptake AND hiv OR hiv AND care OR uptake AND care)	10,647
S 18	S14 OR S15 OR S16 OR S17	113,871
S17	TI ((HIV OR Human AND Immunodeficiency AND virus OR AIDS AND Virus OR AIDS AND Viruses OR acquired AND immune AND deficiency AND syndrome OR Acquired AND Immunodeficiency AND Syndrome OR Human AND Immunodeficiency AND Virus OR Human AND Immunodeficiency AND Viruses OR Acquired AND Immune AND Deficiency AND Syndrome AND Virus)) OR AB ((HIV OR Human AND Immunodeficiency AND virus OR AIDS AND Virus OR AIDS AND Viruses OR acquired AND immune AND deficiency AND syndrome OR Acquired AND Immunodeficiency AND Syndrome OR Human AND Immunodeficiency AND Virus OR Human AND Immunodeficiency AND Viruses OR Acquired AND Immune AND Deficiency AND Syndrome AND Virus)) OR AB ((HIV OR Human AND Immunodeficiency AND virus OR AIDS AND Virus OR AIDS AND Viruses OR acquired AND immune AND deficiency AND syndrome OR Acquired AND Immunodeficiency AND Syndrome OR Human AND Immunodeficiency AND Virus OR Human AND	98,261

	Immunodeficiency AND Viruses OR Acquired AND Immune AND Deficiency AND Syndrome AND Virus))	
S16	SU (HIV OR Human AND Immunodeficiency AND virus OR AIDS AND Virus OR AIDS AND Viruses OR acquired AND immune AND deficiency AND syndrome OR Acquired AND Immunodeficiency AND Syndrome OR Human AND Immunodeficiency AND Virus OR Human AND Immunodeficiency AND Viruses OR Acquired AND Immune AND Deficiency AND Syndrome AND Virus)	62,357
S15	SM (HIV OR Human AND Immunodeficiency AND virus OR AIDS AND Virus OR AIDS AND Viruses OR acquired AND immune AND deficiency AND syndrome OR Acquired AND Immunodeficiency AND Syndrome OR Human AND Immunodeficiency AND Virus OR Human AND Immunodeficiency AND Viruses OR Acquired AND Immune AND Deficiency AND Syndrome AND Virus)	5,450
S 14	KW (HIV OR Human AND Immunodeficiency AND virus OR AIDS AND Virus OR AIDS AND Viruses OR acquired AND immune AND deficiency AND syndrome OR Acquired AND Immunodeficiency AND Syndrome OR Human AND Immunodeficiency AND Virus OR Human AND Immunodeficiency AND Viruses OR Acquired AND Immune AND Deficiency AND Syndrome AND Virus)	60,846
S 13	S9 OR S10 OR S11 OR S12	1,774
S12	TI ((self-test OR self-tests OR self-testing OR self- testers OR self-tested OR self-sampling OR self-examination OR diagnostic self evaluation OR self evaluation OR home test OR home tests OR home testing OR home-based test OR home-based tests OR home-based testing OR self-implemented)) OR AB ((self-test OR self-tests OR self-testing OR self- testers OR self-tested OR self-sampling OR self-examination OR diagnostic self evaluation OR self evaluation OR home test OR home tests OR home testing OR home-based test OR home-based tests OR home-based testing OR self-implemented)) OR AB ((self-test OR self-tests OR self-testing OR self- testers OR self-tested OR self-sampling OR self-examination OR diagnostic self evaluation OR self evaluation OR home test OR home tests OR home testing OR home-based test OR home-based tests OR home-based testing OR self-implemented))	1,527
S11	SU self-test OR self-tests OR self-testing OR self- testers OR self-tested OR self-sampling OR self-examination OR diagnostic self evaluation OR self evaluation OR home test OR home tests OR home testing OR home-based test OR home-based tests OR home-based testing OR self-implemented	548
S10	SM self-test OR self-tests OR self-testing OR self- testers OR self-tested OR self-sampling OR self-examination OR diagnostic self evaluation OR self evaluation OR home test OR home tests OR home testing OR home-based test OR home-based tests OR home-based testing OR self-implemented	4
S 9	KW self-test OR self-tests OR self-testing OR self- testers OR self-tested OR self-sampling OR self-examination OR diagnostic self evaluation OR self evaluation OR home test OR home tests OR home testing OR home-based test OR home-based tests OR home-based testing OR self-implemented	557
S8	S1 OR S2 OR S3 OR S4 OR S5 OR S6 OR S7	120,010
S 7	TI ((adult OR adulthood OR young adult OR young adulthood OR middle –aged)) OR AB (adult OR adulthood OR young adult OR young adulthood OR middle –aged))	48,748
S6	SU young adult	30,939
S5	SM young adult	24
S 4	KW young adult	34,023
S3	SU adult	76,801
S2	SM adult	24
S1	KW adult	81,503

Search in CLINICALTRIALS.GOV		
**Search**	**Query**	**Items**
#1	HIV self test	38
**Search in CROI**		
#1	HIV self -test	36
**Search in IAS CONFERENCE ON HIV PATHOGENESIS, TREATMENT and PREVENTION**		
#1	HIV self -test	150
**Search in HIVST.org**		
#1	HIV self test	160
**Search in OPEN GREY**		
#1	HIV self test*	8
