# Supplementary material for: The effects of HIV self-testing on the uptake of HIV testing, linkage to antiretroviral treatment and social harms among adults in Africa: A systematic review and meta-analysis
Source: PLoS One. 2021 Jan 27;16(1):e0245498. doi: 10.1371/journal.pone.0245498 (PMC7840047; doi:10.1371/journal.pone.0245498)
Supplement: S8 Fig — The RR and horizontal lines represent the 95% CI in fixed-effect model. (DOCX) [file pone.0245498.s010.docx]

**
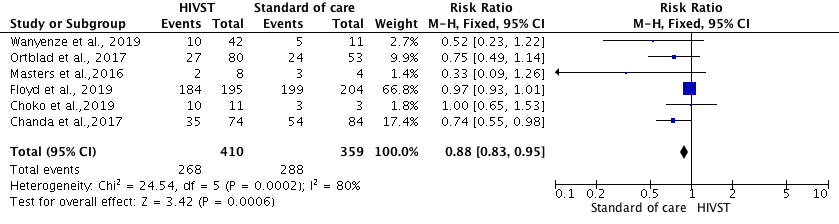
**

**S8 Fig.** Forest plot: Comparison of HIVST versus standard of HIV testing services; outcome: Linkage to HIV care and ART. The RR and horizontal lines represent the 95% CI in fixed-effect model.
